# Danusertib Induces Apoptosis, Cell Cycle Arrest, and Autophagy but Inhibits Epithelial to Mesenchymal Transition Involving PI3K/Akt/mTOR Signaling Pathway in Human Ovarian Cancer Cells

**DOI:** 10.3390/ijms161126018

**Published:** 2015-11-13

**Authors:** Dan Zi, Zhi-Wei Zhou, Ying-Jie Yang, Lin Huang, Zun-Lun Zhou, Shu-Ming He, Zhi-Xu He, Shu-Feng Zhou

**Affiliations:** 1 Department of Obstetrics and Gynecology, Affiliated Hospital of Guizhou Medical University, Guiyang 550004, China; 2 Department of Pharmaceutical Sciences, College of Pharmacy, University of South Florida, Tampa, FL 33612, USA; 3 Department of Gynecologic Oncology Surgery, Affiliated Cancer Hospital of Guizhou Medical University, Guiyang 550002, China; 4 Department of Obstetrics and Gynecology, Xiaolan Hospital, Southern Medical University, Zhongshan 528415, China; 5 Guizhou Provincial Key Laboratory for Regenerative Medicine, Stem Cell and Tissue Engineering Research Center & Sino-US Joint Laboratory for Medical Sciences, Guizhou Medical University, Guiyang 550004, China

**Keywords:** danusertib, ovarian cancer, cell cycle, apoptosis, autophagy, epithelial to mesenchymal transition

## Abstract

Ovarian carcinoma (OC) is one of the most common gynecological malignancies, with a poor prognosis for patients at advanced stage. Danusertib (Danu) is a pan-inhibitor of the Aurora kinases with unclear anticancer effect and underlying mechanisms in OC treatment. This study aimed to examine the cancer cell killing effect and explore the possible mechanisms with a focus on proliferation, cell cycle progression, apoptosis, autophagy, and epithelial to mesenchymal transition (EMT) in human OC cell lines C13 and A2780cp. The results showed that Danu remarkably inhibited cell proliferation, induced apoptosis and autophagy, and suppressed EMT in both cell lines. Danu arrested cells in G_2_/M phase and led to an accumulation of polyploidy through the regulation of the expression key cell cycle modulators. Danu induced mitochondria-dependent apoptosis and autophagy in dose and time-dependent manners. Danu suppressed PI3K/Akt/mTOR signaling pathway, evident from the marked reduction in the phosphorylation of PI3K/Akt/mTOR, contributing to the autophagy inducing effect of Danu in both cell lines. In addition, Danu inhibited EMT. In aggregate, Danu exerts potent inducing effect on cell cycle arrest, apoptosis, and autophagy, but exhibits a marked inhibitory effect on EMT. PI3K/Akt/mTOR signaling pathway contributes, partially, to the cancer cell killing effect of Danu in C13 and A2780cp cells.

## 1. Introduction

Ovarian cancer is the eighth most common type of cancer and the seventh most common cause of cancer-related death in women globally, with 239,000 new cases and around 152,000 deaths in 2012 [1]. In 2015, there has been an estimated 21,290 cases and 14,180 deaths related to ovarian cancer in the US [2], it is the eighth most common cancer and the fifth leading cause of cancer related death, after lung and bronchus, breast, colorectal, and pancreatic cancers in the US [3]. In China, there is an increasing estimated incidence of ovarian cancer [1,4]. The incidence has increased by 30% in the last decade and the mortality rate has increased by 18% with 15,000 deaths each year [1,4]. Clinically, most patients with epithelial ovarian cancer (EOC) are diagnosed at advanced stage with cancer cell metastasis, which substantially jeopardizes the therapeutic opportunity, compromises the therapeutic effect, and increases the risk of recurrence and early death [5]. It has been demonstrated that peritoneal dissemination and ascites are the major causes of patient morbidity and mortality; and currently, platinum- and taxane-based chemotherapy is standard of care for the first-line treatment of patients with advanced ovarian, fallopian tube, or peritoneal cancer [5]. Although responses are observed in up to ~80% of patients; 70%–80% of responding patients eventually will relapse and require further systemic chemotherapy and the five-year survival rate is about 30% to 60% [6]. Patients who relapse >6–12 months after completing platinum-based chemotherapy may respond again to platinum-based therapy, however, it considerably limits the repeated use of platinum agents due to acquired resistance or intolerance in clinical practice [6]. Therefore, there is an urgent need to develop new, safe, and effective therapeutics for the treatment of advanced EOC.

The Aurora kinases belong to the family of highly conserved serine/threonine protein kinases, with an essential role in controlling entry into mitosis, centrosome function, chromosome assembly and segregation [7]. There are three members of the family, including aurora kinase A, B, and C (AURKA/B/C). AURKA is essential for the timely entry into the M phase of the cell cycle, maintaining spindle bipolarity and chromosome segregation. AURKB is required for chromosome condensation, alignment on the spindle, spindle checkpoint function, and cytokinesis. Little is known about the role of AURKC. As the key regulators of mitosis, Aurora kinases are frequently overexpressed in cancer cells and accumulating evidence shows that the aberration in the activity and expression of Aurora kinases has been implicated in the pathogenesis of many types of cancer [8]. Therefore, Aurora kinases have emerged as attractive therapeutic targets for cancer therapies [9,10].

**Figure 1:**
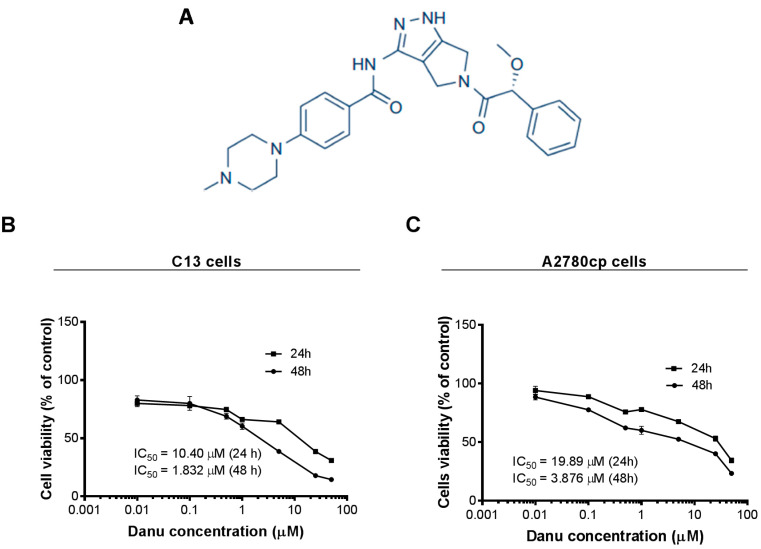
Chemical structure of Danu and cytotoxicity of Danu towards C13 and A2780cp cells. C13 and A2780cp cells were treated with Danu at concentrations ranging from 0.01 to 50 μM for 24 and 48 h. (**A**) Chemical structure of Danu; (**B**) Cell viability of C13 cells; and (**C**) Cell viability of A2780cp cells.

Recently, several small-molecule inhibitors of Aurora kinases have been developed and some of them have shown clinical efficacy in phase I and II clinical trials [11,12]. Among of those, one of the most advanced compounds is Danusertib (Danu, formerly PHA-739358) (Figure 1A), which exerts potent inhibitory activity against AURKA, B, and C with the IC_50_ value of 13, 79, and 61 nM, respectively [13,14,15]. Danu has been investigated in Phase I and II trials, showing a great therapeutic potential in the treatment of a wide range of cancers [14,16]. Moreover, Danu has also shown variable anticancer effect at different stages of preclinical experimental settings [17,18]. However, the effect and underlying mechanisms of Danu have not yet been fully unveiled in ovarian cancer, in particular, under the circumstance of drug resistance. In the present study, we aimed to investigate the effect of Danu on the proliferation, cell cycle distribution, apoptosis, autophagy, and epithelial-to-mesenchymal transition (EMT), and explored the possible mechanisms underlying the anticancer effect of Danu in ovarian cancer cell lines C13 and A2780cp. Notably, these two cell lines are resistant to cisplatin, which often causes therapeutic failure in clinical practice, which emphasizes the importance of the investigation on the effect of Danu in cisplatin resistant cells in the present study.

## 2. Results

### 2.1. Danu Inhibits the Viability of C13 and A2780cp Cells

First, we tested the effect of Danu on the viability of C13 and A2780cp cells using MTT assay. Danu treatment significantly decreased viability at concentrations from 0.01 to 50 μM for 24 and 48 h in both cell lines (Figure 1A,B). In comparison to the control cells, the percentage of the viability for C13 cells was 80.0%–31.0% and 82.9%–14.5% when treated with Danu from 0.01 to 50 μM for 24 and 48 h, respectively (Figure 1B); and the percentage of the viability for A2780cp cells was 94.0%–34.4% and 88.4%–23.4%, when exposed to Danu from 0.01 to 50 μM for 24 and 48 h, respectively (Figure 1C). The IC_50_ values were 10.40 and 1.83 μM for C13 cells after 24- and 48-h incubation with Danu, respectively (Figure 1B). For A2780cp cells, the IC_50_ values were 19.89 and 3.88 μM after 24- and 48-h treatment of Danu, respectively (Figure 1C). Taken together, the results show that Danu exerts a potent inhibitory effect on cell growth in C13 and A2780cp cells.

### 2.2. Danu Induces Cell Cycle Arrest in G_2_/M Phase in C13 and A2780cp Cells

**Figure 2:**
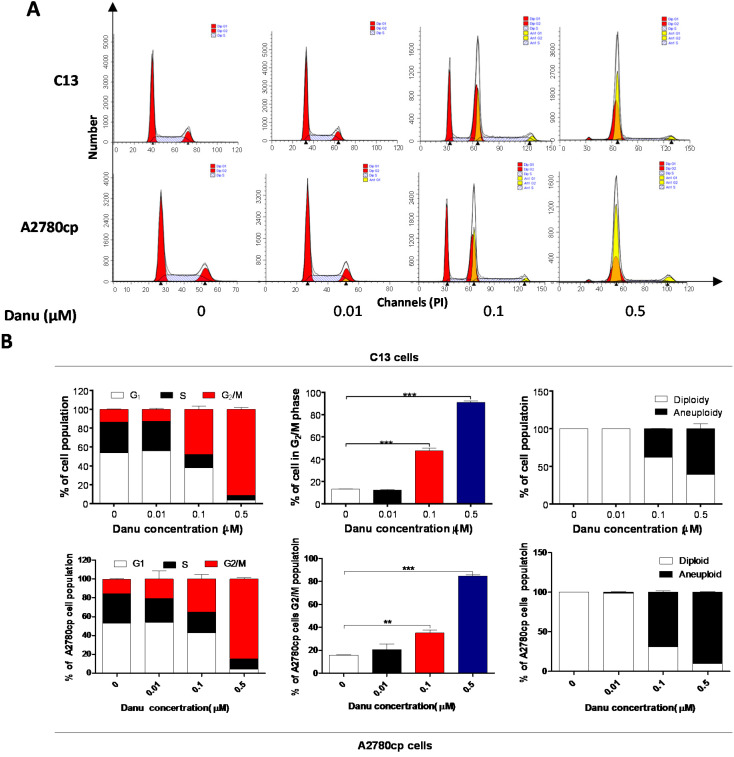
Danu induces cell cycle arrest in G_2_/M phase in C13 and A2780cp cells. Cells were treated with Danu at 0.01, 0.1, and 0.5 μM for 24 h and then subject to flow cytometry. (**A**) Flow cytometric plots of cell cycle distribution of C13 and A2780cp cells and (**B**) bar graphs showing the percentage of C13 and A2780cp cells in G_1,_ S, and G_2_/M phases and the percentage of diploidy and polyploidy in C13 and A2780cp cells. Data represent the mean ± SD of three independent experiments. ** *p* < 0.01 and *** *p* < 0.001 by one-way analysis of variance.

After the observation on the effect of Danu on the viability of C13 and A2780cp cells, we examined the effect of Danu on cell cycle distribution of these two cell lines using flow cytometry. Danu induced a remarkable cell cycle arrest in G_2_/M phase and led to an accumulation of polyploidy in dose dependent manners in C13 and A2780cp cells (Figure 2). Compared to the basal level (13.3%), the percentage of C13 cells arrested in G_2_/M phase ascended to 47.6% and 91.2% when treated with Danu at 0.1 and 0.5 μM for 24 h, respectively (*p* < 0.001, Figure 2A,B). Similarly, in comparison to the control cells (15.6%), the percentage of A2780cp cells arrested in G_2_/M phase was 35.0% and 84.8% when treated with Danu at 0.1 and 0.5 μM, respectively (*p* < 0.001, Figure 2A,B). On the other hand, Danu treatment with increased concentration led to a marked reduction in the number of cells in both G_1_ and S phases (Figure 2A,B). Intriguingly, we observed the accumulation of polyploidy when cells were treated with Danu at 0.1 and 0.5 μM for 24 h, with a 37.7% and 60.5% increase in C13 cells and 69.2% and 90.1% elevation in A2780cp cells, respectively (Figure 2A,B); whereas there was a marked decrease in the percentage of diploidy when treated with Danu at 0.1 and 0.5 μM. The percentage of diploidy decreased from 62.4% to 39.5% in C13 cells and the percentage of diploid decreased from 30.8% to 9.9% in A2780cp cells (Figure 2A,B).

**Figure 3:**
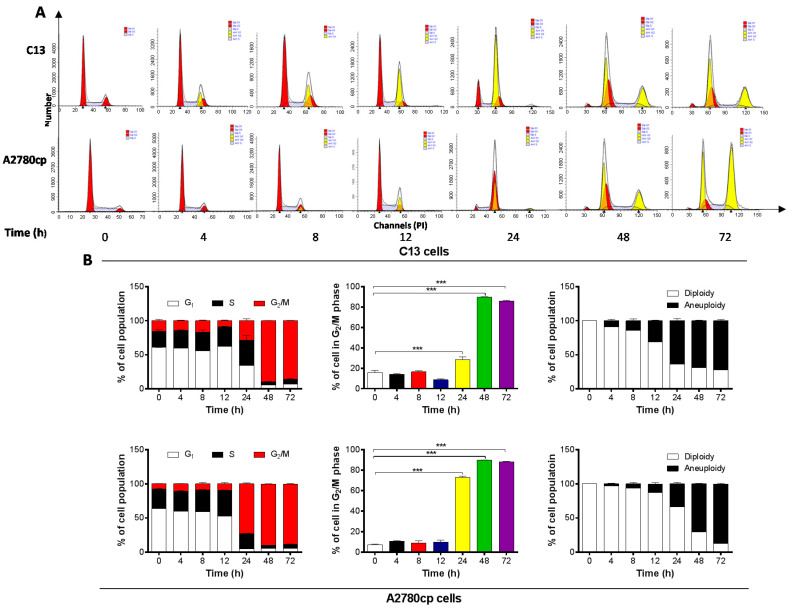
Danu arrests C13 and A2780cp cells in G_2_/M phase over a 72-h treatment period. Cells were treated with 0.5 μM Danu for 4, 8, 12, 24, 48, and 72 h and then subject to flow cytometry. (**A**) Flow cytometric plots of cell cycle distribution of C13 and A2780cp cells and (**B**) bar graphs showing the percentage of C13 and A2780cp cells in G_1_, S, and G_2_/M phases and the percentage of diploidy and polyploidy in C13 and A2780cp cells. Data represent the mean ± SD of three independent experiments. *** *p* < 0.001 by one-way analysis of variance.

To further examine the cell cycle arresting effect of Danu on C13 and A2780cp cells, these two cell lines were treated with 0.5 μM Danu over 72 h. Danu treatment resulted in a marked increase in the percentage of cells arrested in G_2_/M phase and an accumulation of polyploidy in C13 and A2780cp cells (Figure 3A,B). The percentage of C13 cells arrested in G_2_/M phase was increased to 48.7%, 89.7%, and 86.0% from the basal level (15.8%) and the percentage of A2780cp cells arrested in G_2_/M phase was increased to 72.8%, 89.8%, and 88.2% from the basal level (7.2%), when cells were exposed to Danu for 24, 48, and 72 h, respectively (Figure 3A,B). There was also a remarkable reduction in the percentage of cells in both G_1_ and S phases in these two cell lines when treated with Danu treatment for 24, 48, and 72 h (Figure 3A,B). However, there was no alteration in the percentage of cells in G_2_/M phase when C13 and A2780cp cells were incubated with Danu for 4, 8, and 12 h (Figure 3A,B).

Notably, there was an evident occurrence of polyploidy in C13 and A2780cp cells when cells were treated with 0.5 µM Danu from 4 to 72 h. With 4-, 8-, 12-, 24-, 48- to 72-h treatment, the percentage of polyploidy of C13 cells was increased from 8.9%, 14.4%, 31.3%, 63.8%, 68.9% to 72.2%, correspondently, the percentage of diploidy was decreased from 91.1%, 85.6%, 68.7%, 36.2%, 31.1% to 27.8% (Figure 3B). Similarly, the percentage of polyploidy of A2780cp cells was increased from 2.8%, 6.6%, 13.1%, 33.7%, 70.0% to 87.4%, correspondently, the percentage of diploidy was decreased from 7.2%, 93.5%, 86.9%, 66.3%, 30.0% to 12.6% (Figure 3B). Collectively, these results demonstrate that Danu exerts a potent cell cycle arresting effect in C13 and A2780cp cells.

### 2.3. Danu Alters the Expression of Key Cell Cycle Regulators in C13 and A2780cp Cells

**Figure 4:**
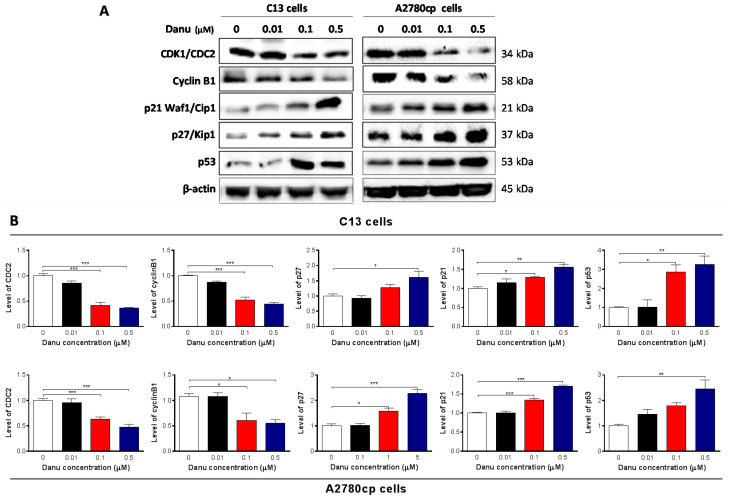
Danu demotes the expression of CDK1/CDC2 and cyclin B1 but promotes the expression of p21 Waf1/Cip1, p27 Kip1, and p53. C13 and A2780cp cells were incubated with Danu 0.01, 0.1, and 0.5 μM for 24 hours and protein samples were subject to Western blotting assays. (**A**) Representative blots of cyclin B1, p21 Waf1/Cip1, p27 Kip1, and p53 and (**B**) bar graphs showing the relative expression level of CDK1/CDC2, cyclin B1, p21 Waf1/Cip1, p27 Kip1, and p53. β-actin was used as the internal control. Data represent the mean ± SD of three independent experiments. * *p* < 0.05, ** *p* < 0.01, and *** *p* < 0.001 by one-way analysis of variance.

We next examined the effect of Danu on the expression of key cell cycle regulators to explain the cell cycle arresting effect, including CDK1/CDC2, cyclin B1, p21 Waf1/Cip1, p27 Kip1, and p53 in C13 and A2780cp cells. Incubation of C13 and A2780cp cells with Danu at 0.01, 0.1, and 0.5 μM resulted in varying alterations in the expression level of key cell cycle regulators (Figure 4). CDC2 and cyclin B1 are two key regulators for G_2_-to-M phase transition [19]. The expression of CDC2 and cyclin B1 was markedly suppressed in both cell lines (Figure 4A,B). There was a 14.3%, 58.4%, and 63.8% reduction in the expression level of CDC2, and 12.8%, 47.4%, and 55.4% decline in the expression level of cyclin B1 in C13 cells, with the treatment of Danu at concentrations of 0.01, 0.1, and 0.5 μM for 24 h, respectively (Figure 4A,B). Similarly, in A2780 cells, there was a 4.1%, 36.9%, and 52.5% decrease in the expression level of CDC2 in A2780cp cells with the treatment of Danu at concentrations of 0.01, 0.1, and 0.5 μM for 24 h, and 43.7%, and 49.1% decrease in the expression level of cyclin B1 with the treatment of Danu at concentrations of 0.1 and 0.5 μM for 24 h, respectively (Figure 4A,B).

Furthermore, p21 Waf1/Cip1, p27 Kip1, and p53 play an important role in the regulation of cell cycle distribution. The tumor suppressor protein p21 Waf1/Cip1 acts as an inhibitor of cell cycle progression, and it serves to inhibit kinase activity and block progression through G_1_/S in association with the CDC2/CDK1-Cyclin B1 complex [20]. Phosphorylated p53 upregulates p21 Waf1/Cip1 transcription via a p53-responsive element, and activation of p53 leads to either cell cycle arrest and DNA repair or apoptosis. The expression of p21 Waf1/Cip1, p27 Kip1, and p53 was significantly increased in both cell lines with the treatment of Danu at concentrations of 0.01, 0.1, and 0.5 μM for 24 h. In comparison to the control cells, incubation of C13 cells with Danu at 0.01, 0.1, and 0.5 μM led to a 1.1-, 1.3- and 1.6-fold increase in the expression level of p21 Waf1/Cip1, respectively (Figure 4A,B). There was a 1.3- and 1.6-fold rise in the expression level of p27 Kip1, and 1.9- and 2.7-fold elevation in the expression level of p53 with the treatment of Danu at 0.1 and 0.5 μM, respectively (Figure 4A,B). There was a similar regulatory effect of Danu on the expression levels of p21 Waf1/Cip1, p27 Kip1, and p53 in A2780cp cells. Treating cells with Danu at 0.1 and 0.5 μM increased the expression level of p21 Waf1/Cip1 1.3- and 1.7-fold, elevated the expression level of p27 Kip1 1.5- and 2.3-fold, and increased the expression level of p53 1.8- and 2.5-fold compared to the control cells, respectively (Figure 4A,B). These results indicate that Danu significantly down-regulates the expression level of CDC2, cyclin B1 but up-regulates the expression level of p21 Waf1/Cip1, p27 Kip1, and p53 in both C13 and A2780cp cells, contributing, at least in part, to the cell cycle arresting effect of Danu in both cell lines.

### 2.4. Danu Induces Apoptosis of C13 and A2780cp Cells via Activation of Mitochondria-Dependent Pathway

**Figure 5:**
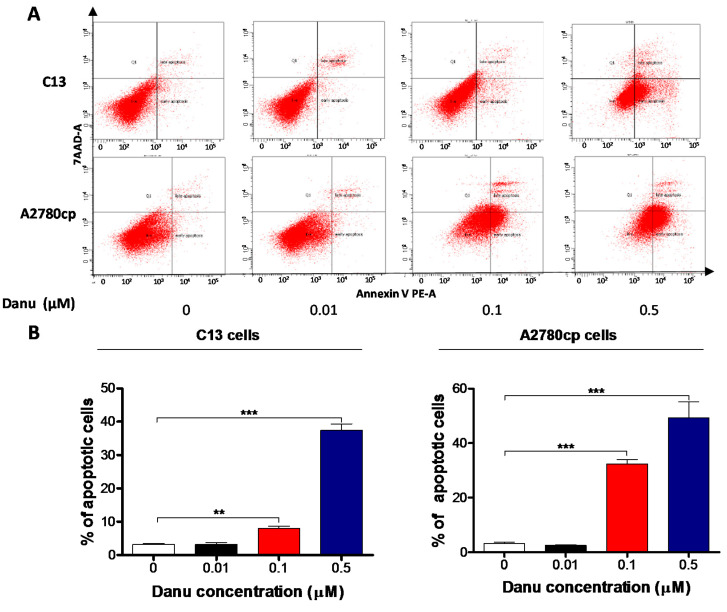
Danu induces apoptotic cell death in C13 and A2780cp cells. C13 and A2780cp cells were incubated with Danu 0.01, 0.1, and 0.5 μM for 48 h and then subject to flow cytometry. (**A**) Flow cytometric plots of specific cell population (live, early apoptosis, and late apoptosis) in C13 and A2780cp cells and (**B**) bar graphs showing the percentage of apoptosis of C13 and A2780cp cells. Data represent the mean ± SD of three independent experiments.** *p* < 0.01 and *** *p* < 0.001 by one-way analysis of variance.

**Figure 6:**
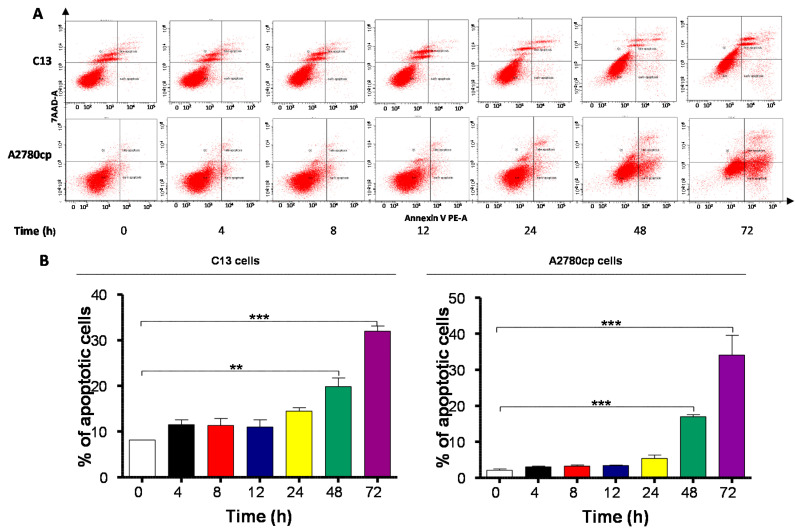
Danu induces apoptotic cell death in C13 and A2780cp cells over a 72-h treatment period. C13 and A2780cp cells were incubated with Danu 0.5 μM for 4, 8, 12, 24, 48, and 72 h and then subject to flow cytometry. (**A**) Flow cytometric plots of specific cell population (live, early apoptosis, and late apoptosis) in C13 and A2780cp cells and (**B**) bar graphs showing the percentage of apoptosis of C13 and A2780cp cells. Data represent the mean ± SD of three independent experiments. ** *p* < 0.01 and *** *p* < 0.001 by one-way analysis of variance.

To further examine the cancer cell killing effect of Danu on C13 and A2780cp cells, the effect of Danu on apoptosis was tested by flow cytometry. Exposure of cells to Danu for 48 h resulted in a remarkable apoptosis of C13 and A2780cp cells (Figure 5 and Figure 6). In C13 cells, the total percentage of apoptotic cells (early + late apoptosis) was 3.2%, 8.0%, and 37.4% when treated with Danu at 0.01, 0.1, and 0.5 μM for 48 h, respectively (Figure 5A,B). There was a 2.6- and 11.9-fold increase when treated with 0.1 and 0.5 μM Danu, compared to the control cells (*p* < 0.001; Figure 5A,B). Treating A2780cp cells with Danu 0.1 and 0.5 μM for 48 h increased the total percentage of apoptotic cells (early and late apoptosis) from 3.2% at the basal level to 32.4% and 49.2%, and there was a 10.1-, and 15.4-fold increase in apoptotic A2780cp cells, compared with the control, respectively (*p* < 0.001, Figure 5A,B). In addition, the effect of Danu on the apoptosis of C13 and A2780cp cells was examined when the cells were treated over 72 h. Incubation of C13 and A2780cp cells with 0.5 μM Danu time-dependently increased the number of apoptotic cells (Figure 6A,B). The percentage of apoptotic C13 cells was increased from 8.2% at basal level (zero time) to 11.6%, 11.4%, 11.0%, 14.5%, 19.9%, and 32.0% when treated with 0.5 μM Danu for 4, 8, 12, 24, 48, and 72 h, respectively; and there was a 2.4- and 3.9-fold rise in the apoptotic C13 cells after 48- and 72-h treatment, respectively (*p* < 0.01 or 0.001; Figure 6A,B). Similarly, the percentage of apoptotic A2780cp cells was increased from 2.2% at basal level to 3.1%, 3.3%, 5.4%, 17.3%, and 34.1% when treated with 0.5 µM Danu for 4, 12, 24, 48 and 72 h, respectively; and there was a 7.9- and 15.5-fold elevation in the apoptotic A2780cp cells after 48- and 72-h treatment, respectively (*p* < 0.001; Figure 6A,B). In aggregate, Danu remarkably induces apoptotic cell death in both C13 and A2780cp cells.

**Figure 7:**
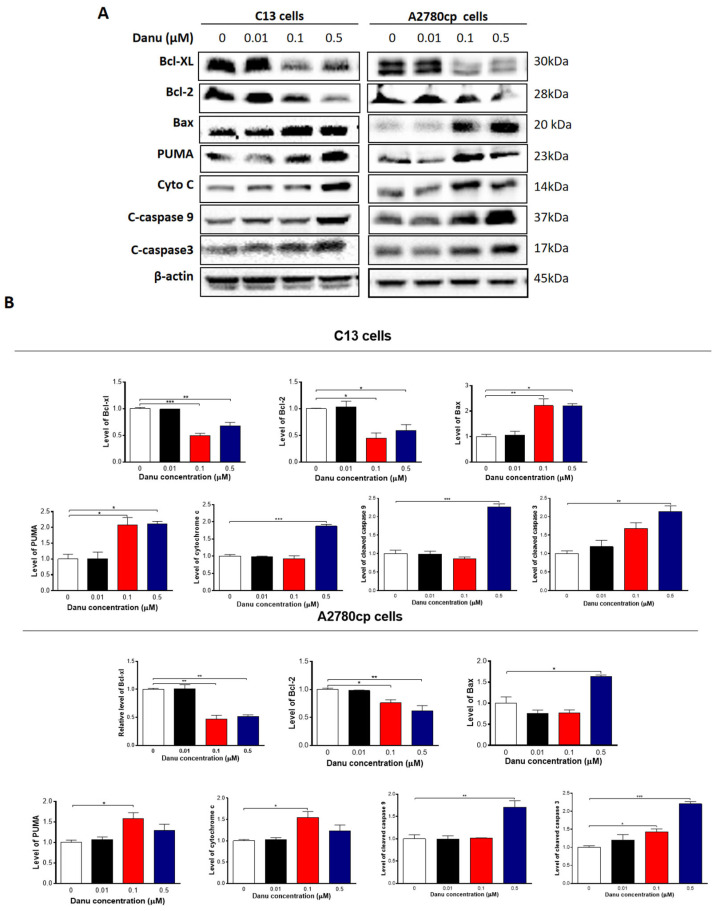
Danu alters the expression of pro-apoptotic and anti-apoptotic proteins in C13 and A2780cp cells. C13 and A2780cp cells were incubated with Danu 0.01, 0.1, and 0.5 μM for 48 h and the protein samples were subject to Western blotting assay. (**A**) Representative blots of B-cell lymphoma-extra-large (Bcl-xl), B-cell lymphoma 2 (Bcl-2), Bcl-2-like protein 4/Bcl-2-associated X protein (Bax), p53 up-regulated modulator of apoptosis (PUMA), cytochrome c, c-caspase 9, and c-caspase 3 in C13 and A2780cp cells. (**B**) Bar graphs showing relative expression level of Bcl-xl, Bcl-2, Bax, PUMA, cytochrome c, c-caspase 9, and c-caspase 3 in C13 and A2780cp cells. β-actin was used as the internal control. Data represent the mean ± SD of the three independent experiments. * *p* < 0.05, ** *p* < 0.01, and *** *p* < 0.001 by one-way analysis of variance. The raw data can be found in the Supplementary Material.

Following the observation of apoptosis-inducing effect of Danu on C13 and A2780 cells, we further examined the effect of Danu on the expression of the key pro-apoptotic and anti-apoptotic proteins in C13 and A2780cp cells to explore the possible mechanisms for the apoptosis-inducing effect of Danu in these two cell lines. As shown in Figure 7A,B, Danu treatment markedly increased the expression level of Bax, while reducing the expression level of Bcl-xl and Bcl-2 in both cell lines. Incubating C13 cells with 0.1 and 0.5 μM Danu for 48 h resulted in a 2.2- and 2.2-fold increase in the expression level of Bax, respectively (*p* < 0.05 or 0.01, Figure 7A,B). In contrast, the expression level of Bcl-xl was decreased 50.9% and 34.3% and the expression level of Bcl-2 was reduced 40.5% and 51.9%, when treated with Danu at 0.1 and 0.5 μM for 48 h, respectively (*p* < 0.05, 0.01, or 0.001, Figure 7A,B). In A2780cp cells, 0.5 μM Danu increased the expression level of Bax 1.5-fold; whereas the expression level of Bcl-xl was decreased 53.4% and 47.5% and the expression level of Bcl-2 was reduced 23.9% and 41. 3% when treated with Danu at 0.1 and 0.5 μM, respectively (*p* < 0.05 or 0.01, Figure 7A,B). Due to the promoting effect of PUMA on apoptosis, the effect of Danu on the expression level of PUMA was also examined. Incubation of C13 cells with 0.1 and 0.5 μM Danu increased the expression level of PUMA 2.0- and 2.1-fold, respectively (*p* < 0.05, Figure 7A,B). In A2780cp cells, the expression level of PUMA was increased 1.6-fold when cells were treated with 0.1 μM Danu (*p* < 0.05, Figure 7A,B).

Disturbance of the balance of pro-apoptotic protein Bax and anti-apoptotic protein Bcl-2 initiates the release of cytochrome c from the mitochondria to the cytosol that is the early event and in turn triggers the activation of the caspase-dependent apoptotic cascade [21]. As such, we examined the effect of Danu treatment on the release of cytochrome c from mitochondria to cytosol in C13 and A2780cp cells. Treatment of these two cell lines with Danu for 48 h markedly increased the cytosolic content of cytochrome c and increased the level of c-caspase 9 and c-caspase 3 (*p* < 0.05, 0.01 or 0.001, Figure 7A,B). Taken together, these results indicate that Danu induces mitochondria-dependent apoptosis in C13 and A2780cp cells.

### 2.5. Danu Induces Autophagy in C13 and A2780cp Cells

**Figure 8:**
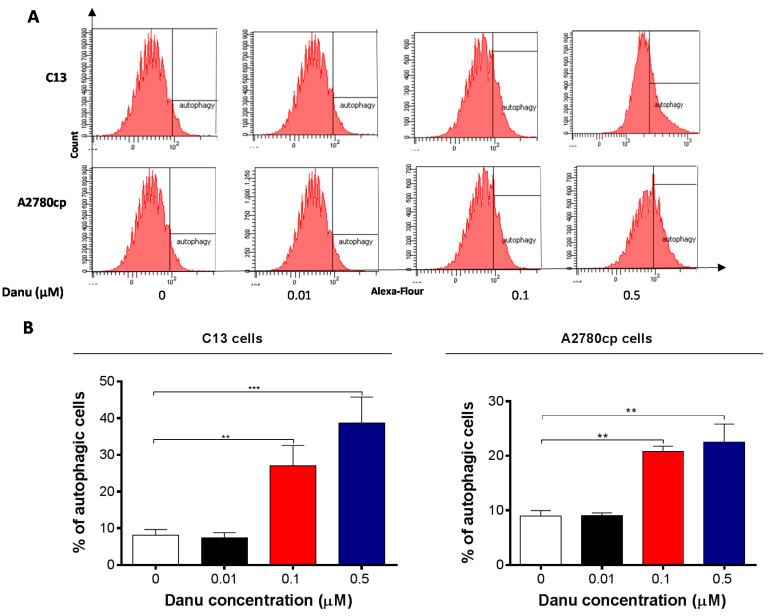
Danu induces autophagy in C13 and A2780cp cells determined by flow cytometry. C13 and A2780cp cells were incubated with Danu 0.01, 0.1, and 0.5 μM for 24 h and the samples were subject to flow cytometry. (**A**) Flow cytometric plots showing autophagy of C13 and A2780cp cells and (**B**) bar graphs showing the percentage of autophagic C13 and A2780cp cells. Data represent the mean ± SD of three independent experiments. ** *p* < 0.01 and *** *p* < 0.001 by one-way analysis of variance.

Next, we also examined the effect of Danu on another dominant type of programmed cell death-autophagy in C13 and A2780cp cells using flow cytometric analysis, confocal fluorescence microscopic examination. As shown in Figure 8A,B, the percentage of autophagy at basal level was 8.0% and 8.9% for C13 and A2780cp cells, respectively. Incubation of C13 and A2780cp cells with Danu for 24 h remarkably increased the percentage of autophagy. In C13 cells, there was a 2.3- and 4.2-fold increase in the percentage of autophagy when treated with 0.1 and 0.5 μM Danu for 24 h compared to the control cells, respectively (*p* < 0.01 or 0.001, Figure 8A,B). Treatment of A2780cp cells with 0.1 and 0.5 μM Danu for 24 h resulted in a 1.6- and 1.8-fold increase in the percentage of autophagy, respectively (*p* < 0.01, Figure 8A,B). Low concentration of Danu (0.01 μM) did not significantly induce autophagy in both cell lines (Figure 8A,B).

**Figure 9:**
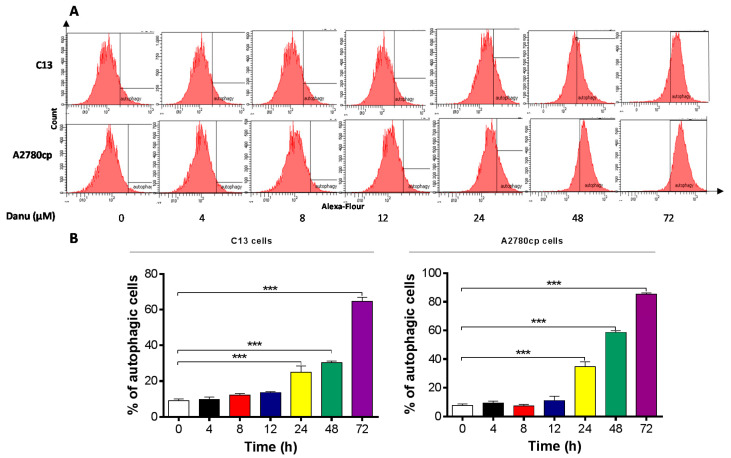
Danu induces autophagy in C13 and A2780cp cells over a 72-h treatment period determined by flow cytometry. C13 and A2780cp cells were incubated with Danu 0.5 μM for 4, 8, 12, 24, 48, and 72 h and the samples were subject to flow cytometry. (**A**) Flow cytometric plots showing autophagy of C13 and A2780cp cells and (**B**) bar graphs showing the percentage of autophagic C13 and A2780cp cells. Data represent the mean ± SD of three independent experiments. *** *p* < 0.001 by one-way analysis of variance.

In separate experiments, the autophagic effect of Danu in C13 and A2780cp cells was examined when cells were treated over 72 h. Danu treatment induced a time-dependent increase in autophagy in both cell lines (Figure 9A,B). In C13 cells, the percentage of autophagy was increased from 9.0% at basal level (zero time) to 9.7%, 12.1%, 13.6%, 25.0%, 30.4%, and 64.7% when treated with Danu for 4, 8, 12, 24, 48, and 72 h, respectively (Figure 9A,B). Treatment of A2780cp cells with 0.5 μM Danu increased the percentage of autophagy from 7.5% at basal level (zero time) to 9.3%, 7.3%, 11.2%, 34.7%, 58.7%, and 85.4% when treated with Danu for 4, 8, 12, 24, 48, and 72 h, respectively (Figure 9A,B).

**Figure 10:**
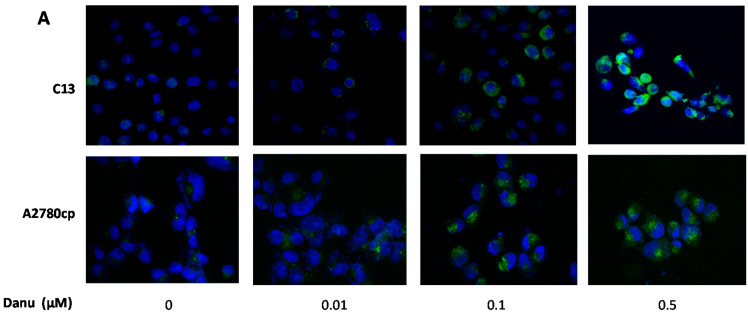

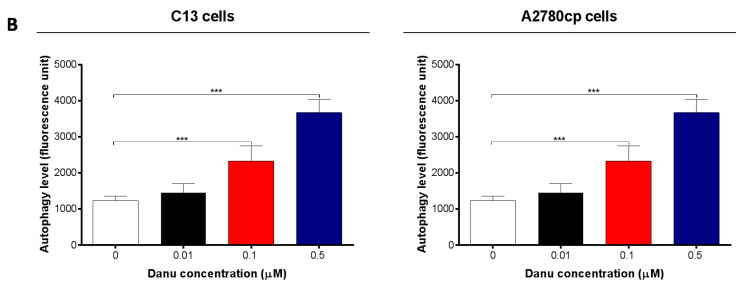
Danu induces autophagy in C13 and A2780cp cells determined by confocal microscopy. C13 and A2780cp cells were incubated with Danu 0.01, 0.1, and 0.5 μM for 24 h and then subject to confocal microscopy. (**A**) Representative images showing autophagy of C13 and A2780cp cells and (**B**) bar graphs showing the percentage of autophagic C13 and A2780cp cells. Data represent the mean ± SD of three independent experiments. Magnification: 60×; scale bar: 5 μm. *** *p* < 0.001 by one-way analysis of variance.

Further, the autophagy-inducing effect of Danu in C13 and A2780cp cells was examined using confocal microscopy. In comparison to the control cells, Danu treatment concentration-dependently increased the autophagy in C13 and A2780cp cells (Figure 10A,B). There was a 1.8- and 2.7-fold increase in the autophagy of C13 cells when treated with Danu at 0.1 and 0.5μM for 24 h, respectively (*p* < 0.001, Figure 10A,B). In A2780cp cells, there was a 1.6- and 2.6-fold elevation in autophagy when treated with 0.1 and 0.5 μM Danu, respectively (*p* < 0.001, Figure 10A,B). Taken together, these results demonstrate that Danu exerts a potent autophagy-inducing effect in C13 and A2780cp cells.

### 2.6. Danu Suppresses the PI3K/Akt/mTOR Signaling Pathway in C13 and A2780cp Cells

**Figure 11:**
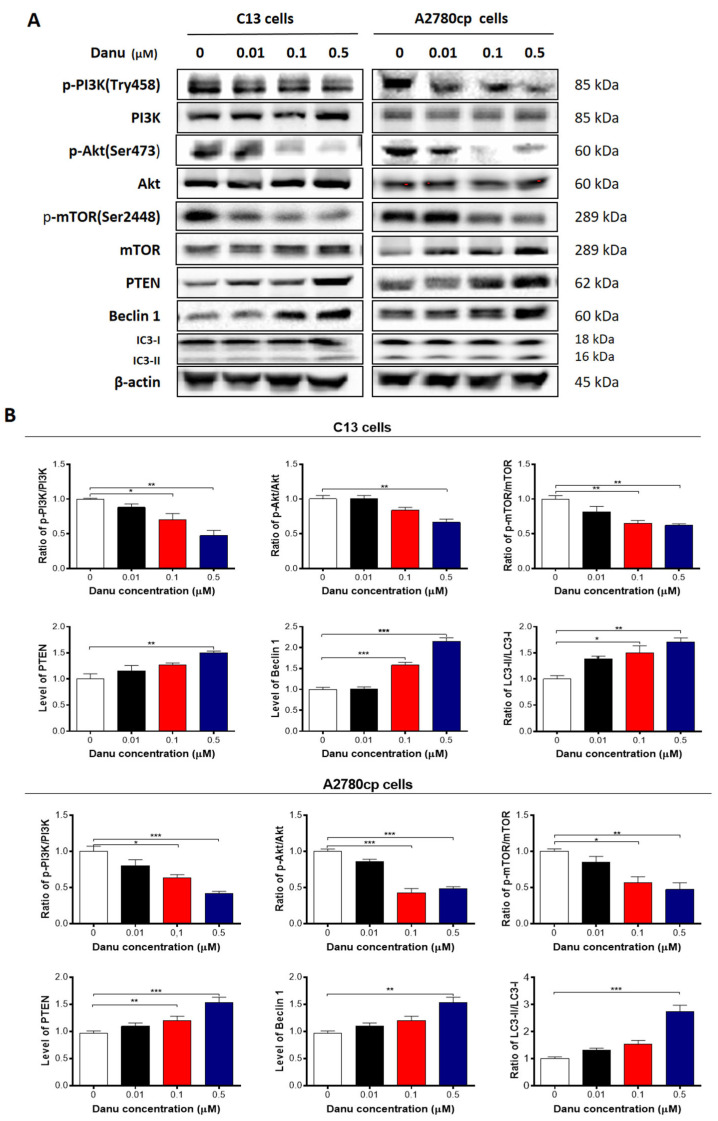
Danu alters the expression of pro-autophagic and anti-autophagic proteins in C13 and A2780cp cells. C13 and A2780cp cells were incubated with Danu 0.01, 0.1, and 0.5 μM for 24 h and the protein samples were subject to Western blotting assay. (**A**) Representative blots of phosphorylation level of PI3K, Akt, and mTOR, and the total level of PI3K, Akt, mTOR, PTEN, beclin 1, LC 3-I, and LC 3-II in C13 and A2780cp cells. (**B**) Bar graphs showing the ratio of p-PI3K/PI3K, p-Akt/Akt, and p-mTOR/mTOR, and expression of PTEN, beclin 1, LC 3-I, and LC 3-II in C13 and A2780cp cells. β-actin was used as the internal control. Data represent the mean ± SD of the three independent experiments. * *p* < 0.05, ** *p* < 0.01, and *** *p* < 0.001 by one-way analysis of variance. The raw data can be found in the Supplementary Material.

Next, we explored the possible mechanisms for the autophagy-inducing effect of Danu in C13 and A2780cp cells. PI3K/Akt/mTOR signaling is the major pathway involved in autophagy [22]. We initially examined the phosphorylation of PI3K at Tyr458. In comparison to the control cells, Danu treatment markedly inhibited the phosphorylation of PI3K at Tyr458 rather than the expression level of PI3K when these two cell lines were treated with Danu at 0.1 and 0.5 µM. Consequently, the p-PI3K/PI3K ratio was decreased in C13 and A2780cp cells in a concentration-dependent manner in response to Danu treatment (Figure 11A,B). There was a 30.0% and 52.6% decrease in p-PI3K/PI3K ratio when C13 cells were treated with Danu 0.1 and 0.5 μM for 24 h compared to the control, respectively (*p* < 0.05 or 0.01; Figure 11A,B). In A2780cp cells, the ratio of p-PI3K over PI3K was decreased by 36.6% and 58.1% in response to 0.1 and 0.5 μM Danu treatment, compared with the control, respectively (*p* < 0.05 or 0.001; Figure 11A,B). Having determined the regulatory effect of Danu on PI3K, we next evaluated the effect of Danu on phosphorylation of Akt at Ser473 and mTOR at Ser2448 and the expression of phosphatase and tensin homolog (PTEN) in C13 and A2780cp cells (Figure 11A,B). As a downstream effector of PI3K, Akt is involved in regulation of various signaling pathways involved in cell metabolism, proliferation, survival, growth, and angiogenesis [23]. Danu inhibited the activation of Akt at Ser473 without a significant change in the expression level of Akt in either cell line (Figure 11A,B). The ratio of p-Akt over Akt was decreased 37.2% when C13 cells were treated with 0.5 μM Danu for 24 h, compared with control cells (*p* < 0.01, Figure 11A,B). Similarly, the ratio of p-Akt over Akt was decreased by 57.4% and 51.3% in A2780cp cells when treated with 0.1 and 0.5 μM Danu, respectively (*p* < 0.001, Figure 11A,B). The expression level of PTEN that leads to the constitutive activation of downstream components of the PI3K pathway in cancer cells, was markedly increased in C13 and A2780cp cells when treated with Danu (Figure 11A,B). In addition, exposure of these two cell lines to Danu resulted in a marked decrease in the phosphorylation of mTOR at Ser2448, whereas there was no significant change in expression of mTOR in these two cell lines. The ratio of p-mTOR over mTOR was decreased 34.9% and 38.1% when C13 cells were treated with Danu at 0.1 and 0.5 μM for 24 h, respectively (*p* < 0.01, Figure 11A,B). In A2780cp cells, the p-mTOR/mTOR ratio was decreased 43.0% and 52.5% in response to Danu treatment at 0.1 and 0.5 μM for 24 h, respectively (*p* < 0.05 or 0.01, Figure 11A,B).

Next, we evaluated the effect of Danu on expression of beclin 1, LC3-I, and LC3-II in C13 and A2780cp cells which are important markers of vesicle expansion and formation during the autophagy process. Treating the cells with Danu significantly increased the expression level of beclin 1 and conversion of LC3-I to LC3-II in C13 and A2780cp cells (Figure 11A,B). There was a 1.6- and 2.2-fold increase in expression level of beclin 1 in C13 cells treated with 0.1 and 0.5 μM Danu for 24 h, respectively (*p* < 0.001, Figure 11A,B). In A2780cp cells, there was a 1.5-fold increase in expression level of beclin 1 in response to 0.5 μM Danu treatment for 24 h (*p* < 0.01, Figure 11A,B). In addition, there was a marked increase in the expression level of LC3-II in both C13 and A2780cp cells (Figure 11A,B). Compared with control cells, there was a 1.5- and 1.7-fold increase in the ratio of LC3-II/LC3-I when C13 cells were treated with 0.1 and 0.5 μM Danu for 24 h, respectively (*p* < 0.05 or 0.01, Figure 11A,B). In A2780cp cells, treatment with 0.1 and 0.5 μM Danu resulted in a 2.6-fold increase in the ratio of LC3-II/LC3-I (*p* < 0.001, Figure 11A,B). Collectively, these findings indicate that inactivation of PI3K/Akt/mTOR signaling pathway contributes to Danu-induced autophagy in C13 and A2780cp cells.

### 2.7. PI3K/Akt/mTOR Pathway Inhibitors Contribute to Danu-Induced Autophagy in C13 and A2780cp Cells

**Figure 12:**
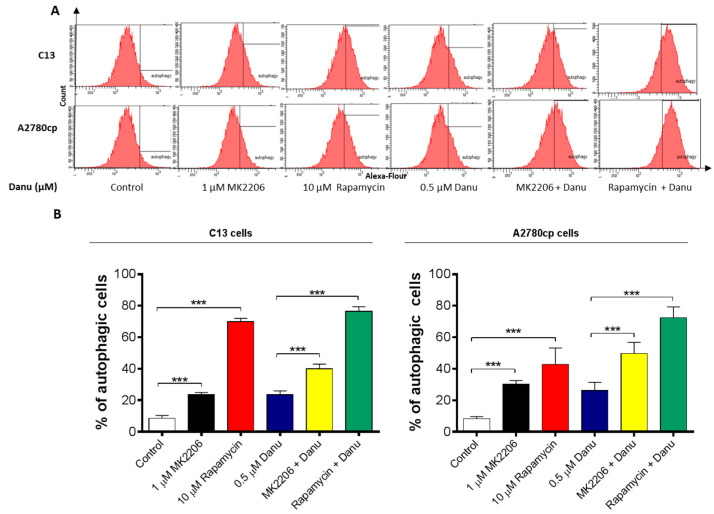
Danu induces autophagy with the involvement of PI3K/Akt/mTOR signaling pathway. C13 and A2780cp cells were pretreated with MK-2206 (1 μM) or rapamycin (10 μM) for 1 hour and then co-treated with 0.5 µM Danu for 24 h. The samples were subject to flow cytometric analysis. (**A**) Histograms show autophagy of C13 and A2780cp cells. (**B**) Bar graph showing the percentage of autophagic C13 and A2780cp cells. Data are the mean ± SD of three independent experiments. *** *p* < 0.001 by one-way analysis of variance.

Since we have observed regulatory effect of Danu on PI3K/Akt/mTOR pathway in both C13 and A2780cp cells, we then employed specific chemical inhibitors (MK-2206, an inhibitor of Akt and rapamycin, an inhibitor of mTOR) to further validate the role of this signaling pathway in Danu-induced autophagy. As shown in Figure 12A,B, MK-2206 (1 μM) or rapamycin (10 μM) markedly induced autophagy in C13 and A2780cp cells; compared to the control, incubation of cells with MK2206 and rapamycin increased 2.8- and 8.2-fold in the autophagy of C13 cells and 3.6- and 5.1-fold in the autophagy of A2780cp cells, respectively (*p* < 0.001, Figure 12A,B). Notably, co-incubation of MK-2206 (1 μM) or rapamycin (10 μM) with Danu enhanced Danu-induced autophagy (Figure 12). Compared to Danu treated cells, co-treatment of C13 cells with Danu and MK2206 or rapamycin increased 1.2- and 3.3-fold in autophagy cells, respectively (*p* < 0.001, Figure 12A,B). In A2780cp cells, co-incubation cells with Danu and MK2206 or rapamycin led to a 1.9- and 2.8-fold increase in the autophagic cells compared to Danu treated cells (*p* < 0.001, Figure 12A,B). Taken together, the results suggest that Danu-induced autophagy may be, at least in part, ascribed to the regulation of PI3K/Akt/mTOR signaling pathway.

### 2.8. Danu Inhibits EMT Phenotype in C13 and A2780cp Cells

**Figure 13:**
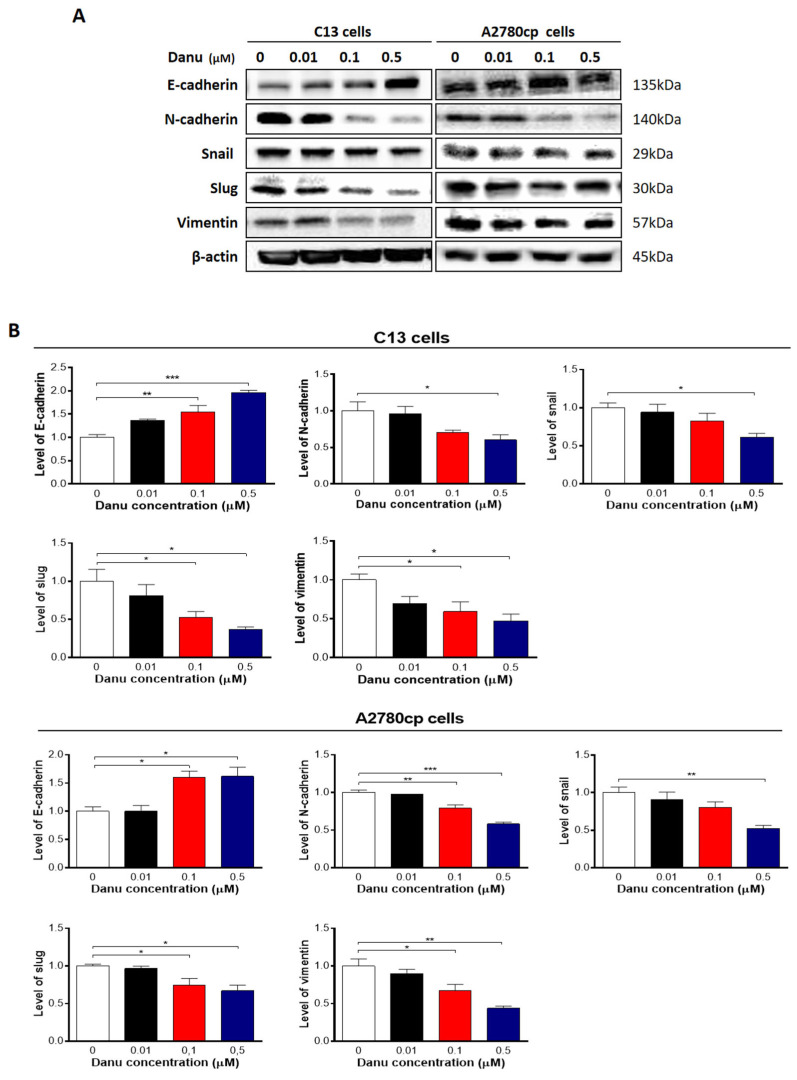
Danu inhibits EMT in C13 and A2780cp cells. C13 and A2780cp cells were incubated with Danu 0.01, 0.1, and 0.5 μM for 24 h and the protein samples were subject to Western blotting assay. (**A**) Representative blots showing E-cadherin, N-cadherin, snail, slug, and vimentin levels in C13 and A2780cp cells. (**B**) Bar graphs showing the relative expression of E-cadherin, N-cadherin, snail, slug, and vimentin in C13 and A2780cp cells. β-actin was used as the internal control. Data represent the mean ± SD of the three independent experiments. * *p* < 0.05, ** *p* < 0.01, and *** *p* < 0.001 by one-way analysis of variance. The raw data can be found in the Supplementary Material.

EMT is a critical process playing an important role in cancer invasion and metastasis [24]. Reduction in expression of epithelial markers drives EMT with the involvement of a number of key functional proteins, such as E-cadherin, while other mesenchymal markers and transcription factors, such as *N*-cadherin, snail, slug, and, vimentin, are upregulated in EMT [24]. Herein, we examined the effect of Danu on EMT-associated key functional proteins in C13 and A2780cp cells using Western blotting assays. As shown in Figure 13A,B, incubation of C13 cells with Danu resulted in a marked increase in expression level of E-cadherin and a decrease in expression level of *N*-cadherin. In comparison with the control cells, there was a 1.6- and 2.0-fold increase in the level of *E*-cadherin when C13 cells were treated with Danu at 0.1 and 0.5 μM, respectively (*p* < 0.05 or 0.01, Figure 13A,B); and there was a 39.7% decrease in level of *N*-cadherin when C13 cells were treated with 0.5 μM Danu (*p* < 0.05, Figure 13A,B). In A2780cp cells, there was a 1.6- and 1.6-fold increase in level of E-cadherin in response to 0.1 and 0.5 μM Danu treatment, respectively (*p* < 0.05, Figure 13A,B); and the level of *N*-cadherin was decreased 21.1% and 41.9% when treated with Danu at 0.1 and 0.5 μM, respectively (*p* < 0.05 or 0.01, Figure 13A,B). Consequently, Danu treatment enhanced the epithelial features in both cell lines, evident from the marked increase in the ratio of E-cadherin over *N*-cadherin.

We next examined the expression of snail and slug, which are suppressors of E-cadherin. As shown in Figure 13A,B, Danu concentration-dependently reduced the expression level of snail and slug in both cell lines. In C13 cells, Danu treatment at 0.1 and 0.5 μM resulted in a 47.4% and 63.5% reduction in the expression level of slug, respectively, compared with control cells (*p* < 0.05, Figure 13A,B), and 0.5 μM Danu caused a 39.0% decrease in the expression level of snail when compared with control cells (*p* < 0.05, Figure 13A,B). There was a similarly effect of Danu in A2780cp cells. In comparison with the control cells, there was a 25.5% and 32.9% decrease in the level of slug when treated with 0.1 and 0.5 μM Danu, respectively; and there was a 47.5% decrease in the level of snail when treated with 0.5 μM Danu (*p* < 0.05 or 0.01, Figure 13A,B). Moreover, there was a concentration-dependent reduction in the level of vimentin in C13 and A2780cp cells when exposed to Danu for 24 h (Figure 13A,B). Treatment of cells with Danu at 0.1 and 0.5 µM led to 41.0% and 53.1% decrease in the level of vimentin in C13 cells and 33.1% and 56.1% decline in A2780cp cells, respectively (*p* < 0.05 or 0.01, Figure 13A,B). Taken together, Danu possesses a potent inhibitory effect on EMT evident from the concentration-dependent regulatory effect on EMT-associated markers in C13 and A2780cp cells.

## 3. Discussion

Ovarian cancer has the highest fatality to case ratio of all gynecological cancers because the majority of cases are diagnosed at late stage. Despite of significant effort to improve the early detection and advances in chemotherapy, clinical management of ovarian cancer remains a major challenge to human health [25,26,27]. The standard treatment for advanced OC includes both cytoreductive surgery and platinum-based chemotherapy [28,29]. Initial response rates are high, within a 3-year period, although most (~70%) patients experience recurrence, ultimately, the development of chemoresistance [30,31]. Improved understanding of the tumor biology led to the development of targeted anticancer medications that have the ability to improve tumor responses to chemotherapy, thereby alleviating some of the limitations of the chemotherapy [32,33]. The more advanced agent in this regard is Danu, a small adenosine triphosphate competitive molecule that inhibits AURKA/B/C, which has entered Phase II clinical trials for patients with solid tumors [14]. Danu exhibits potent inhibitory effect on AURKB activity, as determined by inhibition of its substrate histone H3 in position Ser10 and also on AURKA, as determined by inhibition of autophosphorylation in position Thr288 [34,35]. It has been shown that Danu inhibits the proliferation of gastric cancer and breast cancer cells via inducing cell cycle arrest and programmed cell death and inhibits EMT [17,18]. In the present study, our findings have shown a potent inhibitory effect of Danu on cell proliferation in two ovarian cancer cell lines C13 and A278cp cells. The findings indicate that Danu induces cell cycle arrest and promotes cell apoptosis and autophagy, with the involvement of PI3K/Akt/mTOR signaling pathway in both of cell lines. Additionally, the results suggest that Danu also inhibits EMT contributing to the anticancer effect in both cell lines.

AURKA, B, and C are a family of serine/threonine kinases with the highest degree of sequence homology in their catalytic domains [8,36]. Enhanced activity or expression of aurora kinases has been observed in different human malignancies [37,38]. Recently, a number of Aurora kinase inhibitors have been discovered and are under preclinical or clinical experimental settings. Danu, a pan-aurora kinases inhibitor, has the inhibitory effect on the proliferation of hepatocellular carcinoma, pancreatic cancer, gastric, and breast cancer cells via inducing cell division failure and resulting in polyploidy [17,18,39,40]; and Danu has been under Phase II clinical trials for patients with solid tumors [14].

Our study found that cell cycle transition from G_2_ phase to M phase was inhibited by Danu in C13 and A278cp cell lines. It has been firstly reported that the Danu induced cell cycle arrest in G_2_/M phase in cisplatin resistant C13 and A2780cp cells. G_2_/M transition is controlled by CDC2, also known as CDK1, which partners with cyclin B1. CDC family proteins and cyclin complexes play a critical role in the regulation of G_2_/M transitions [20]. We further found that the cell cycle checkpoints regulators including CDC2/CDK1 and cyclin B1 were involved. This is evident from a remarkable reduction in the expression level of CDC2/CDK1 and cyclin B1 after Danu treatment, which is associated with G_2_/M cell cycle arrest. Moreover, the inhibition of cyclin–CDK complex might be amplified by simultaneous up-regulation of the p21Waf1/Cip1 protein that is a CDK inhibitor [41]. Activated p53 can inhibit cell cycle progression by regulation of p21Waf1/Cip1 [42]. Our findings showed that Danu treatment increased the level of p21Waf1/Cip1 and p53 in both cell lines, which probably contributes to the inhibitory effect of Danu on cell proliferation and inducing effect on cell cycle arrest in G_2_/M phase in C13 and A2780cp cells.

Apoptosis, a tightly regulated signaling process that involves the coordination of both anti-apoptotic and pro-apoptotic proteins, is vital for anti-carcinogenesis [43]. Increasing evidence shows that manipulating apoptosis by modulation of the key regulators of apoptosis is a promising therapeutic strategy in the treatment of cancer [44,45]. The Bcl-2 family members, such as Bcl-xl, Bcl-2 and Bax, are essential for the initiation of mitochondrial dysfunction during apoptotic process. Bcl-2 can be inhibited by post-translational modification and/or by increased expression of PUMA, which is an essential regulator of p53-mediated cell apoptosis [45]. In the present study, we observed concentration- and time-dependent pro-apoptotic effect of Danu on C13 and A2780cp cells. The findings showed that the expression of pro-apoptotic factor Bax was markedly enhanced in Danu treatment group. However, the expression of anti-apoptotic factor Bcl-xl and Bcl-2 was inhibited significantly. Also, Danu treatment led to an increase in the level of cleaved caspase-3 and caspase-9. Danu could also induce release of cytochrome c from mitochondria to cytosol in C13 and A2780cp cells. These results indicate that Danu could induce apoptosis in C13 and A2780cp cells by activating the mitochondrial apoptosis pathway.

Autophagy is an evolutionarily conserved catabolic pathway where cells deliver their own cytoplasmic material and/or organelles to lysosomes for degradation [46]. It is an important type of programmed cell death involving the engulfment and degradation of nonessential or abnormal cellular organelles and proteins in living cells [47,48]. This autophagy cells via a lysosomal catabolic pathway for engulfment, degradation, and recycling of nonessential or abnormal cellular organelles and proteins in living cells. This complicated process is executed through multiple steps from intracellular membrane/vesicle reorganization to form double-membraned autophagosomes that fuse with lysosomes to form autophagolysomes, which degrade the contents via acidic lysosomal hydrolases [6,49]. Recently, the role of autophagy in the treatment of cancer has been debated. We observed that Danu-induced autophagy in dose- and time-dependent manners in the present study. This could be regarded as one of the anticancer effects, although more studies are needed to further test this autophagy-inducing effect in the treatment of ovarian cancer. The PI3K/Akt/mTOR signaling pathway is a central pathway involved in autophagy through the regulation of cell growth, motility, protein synthesis, cell metabolism, cell survival, and cell death in response to various stimuli [50]. The phosphorylation of Akt would be up-regulated following the activation of PI3K and mTOR can integrate upstream activating signals through PI3K/Akt pathway and become phosphorylated, and then inhibits autophagy [51]. In our study, Danu can down-regulate the phosphorylation level of PI3K, Akt, and mTOR, while promoting the expression of PTEN, as a regulator of the PI3K/Akt/mTOR pathway. Rapamycin is allosteric inhibitors of mTOR via their binding to FKBP12, and were among the first mTOR-targeted therapeutics to enter the clinic [52]. A number of studies have shown that rapamycin induce feedback activation of Akt and the combination of rapamycin and Akt inhibitors results in additive or synergistic antitumor effects [53,54,55]. MK2206, a potent, irreversible, and selective Akt inhibitor and a blocker of autophagosome formation, markedly increased the Danu-induced autophagic cell death in both C13 and A2780cp cells. We combined the treatment of Danu with MK-2206 and rapamycin respectively, which resulted an enhanced effect on Danu-induced autophagy. Thus, the findings suggest that Danu induces autophagy via suppressing the PI3K/Akt/mTOR pathway in both C13 and A2780cp cells. Taken together, Danu-induced apoptosis and autophagy contributes to the cancer cell killing effect on C13 and A2780cp cells. Notably, the present study showed a higher IC_50_ value of Danu towards the cisplatin resistant A2780cp cells than that in A2780 cells [13]. Moreover, Danu exhibited a more potent cancer cell killing effect evident from the lower IC_50_ values in previous studies in different cancer cell lines [17,18]. The findings suggest that drug resistance affect the effect of Danu in C13 and A2780cp cells.

The ability of epithelial cells to undergo mesenchymal transition during embryogenesis, wound healing and malignant tumor progression is now widely accepted as a core biological process termed the EMT [24,56]. Tumor cells shed their differentiated epithelial characteristics, in order to invade and metastasize, including cell–cell adhesion and polarity, and acquire mesenchymal traits, such as invasiveness, motility and, importantly, many of the attributes of stem cells [57,58]. In the metastasis of cancer, the increased motility and invasive behavior of cancer cells underwent EMT, due to the loss of adhesion molecules such as E-cadherin, and the gain of mesenchymal proteins such as *N*-cadherin and vimentin [59]. In this study, Danu significantly promoted the expression of E-cadherin; however, it demoted the expression of N-cadherin, leading to an EMT inhibition in ovarian cancer cell. Danu suppressed the expression of snail and slug, in C13 and A2780cp cells, which provides an explanation for the inhibitory effect of Danu on EMT. Snail and slug are master regulators of EMT, down-regulating *E*-cadherin by silencing gene expression. Furthermore, Danu decreased the expression level of vimentin. Vimentin is a marker expressed in mesenchymal cells [60,61]. Collectively, these results suggest that Danu could inhibit EMT in C13 and A2780cp cells.

In conclusion, Danu inhibits cell proliferation, induces cell-cycle arrest, mitochondria-dependent apoptosis and autophagy in C13 and A2780cp cells. Inhibition of PI3K/Akt/mTOR signaling pathway is involved in the autophagy-inducing effect of Danu in C13 and A2780cp cells. Suppression of EMT may render Danu as a promising agent to curb cancer metastasis in patients at advanced stage. Danu may represent a new anticancer drug that can kill ovarian cancer cells and prevent metastasis. More functional and mechanistic studies are warranted to elucidate the role of Danu in the treatment of human ovarian carcinoma.

## 4. Materials and Methods

### 4.1. Chemicals and Reagents

4,6-Diamidino-2-phenylindole was obtained from Invitrogen Inc. (Carlsbad, CA, USA). MK-2206 (a selective inhibitor of protein Kinase B, (Akt)) and rapamycin (a specific inhibitor of mammalian target of rapamycin, (mTOR)) were purchased from Selleckchem Inc. (Houston, TX, USA). Dulbecco’s Modified Eagle’s Medium (DMEM) was sourced from Corning Cellgro Inc. (Herndon, VA, USA). Dulbecco’s phosphate-buffered saline (D-PBS), fetal bovine serum (FBS), phosphatase and protease inhibitor cocktails, propidium iodide (PI), 4-(2-hydroxyethyl) piperazine-1-ethanesulfonic acid (HEPES), ethylenediaminetetraacetic acid, RNase A, and thiazolyl blue tetrazo-lium bromide (MTT) were purchased from Sigma-Aldrich (St. Louis, MO, USA). The Annexin V: phycoerythrin (PE) apoptosis detection kit was bought from BD Biosciences Inc. (San Jose, CA, USA) and the Cyto-ID^®^ autophagy detection kit was obtained from Enzo Life Sciences Inc. (Farmingdale, NY, USA). The Pierce bicinchoninic acid (BCA) protein assay kit, skim milk, and Western blotting substrate were purchased from Thermo Scientific Inc. (Hudson, NH, USA). A polyvinylidene difluoride (PVDF) membrane was obtained from Bio-Rad Inc. (Hercules, CA, USA). Primary antibodies against human cyclin B1, cyclin-dependent kinase 1 (CDK1/CDC2/CDKN1), p21 Waf1/Cip1, p27 Kip1, p53, cytochrome c, Bcl-2-like protein 4/Bcl-2-associated X protein (Bax), B-cell lymphoma-extra large (Bcl-xl), B-cell lymphoma 2 (Bcl-2), cleaved (c-) caspase 9, c-caspase 3, p53 up-regulated modulator of apoptosis (PUMA), phosphatidylinositol 3-kinase (PI3K), phosphorylated (p-) PI3K/p85 at Tyr458, Akt, p-Akt at Ser473, mTOR, p-mTOR at Ser2448, PTEN, beclin 1, microtubule-associated protein 1A/1B-light chain 3 (LC3-I), LC3-II, *E*-cadherin, *N*-cadherin, vimentin, slug, and snail were all purchased from Cell Signaling Technology Inc. (Beverly, MA, USA). The antibody against human β-actin was sourced from Santa Cruz Biotechnology Inc. (Dallas, TX, USA).

### 4.2. Cell Lines and Cell Culture

C13 and A2780cp cells are human EOC cell lines that were obtained from Chengxiong Xu (Moffitt Cancer Center & Research Institute, Tampa, FL, USA) and were cultured in DMEM media. These two cell lines are epithelial ovarian cancer cell lines and belong to high-grade serous carcinoma. They exhibit similar biological behavior regarding the characteristics of aggressiveness, invasiveness, and metastasis. All media containing l-glutamine, phenol red, l-cysteine and l-methionine, sodium bicarbonate were supplemented with 10% heat-inactivated FBS. The cells were maintained at 37 °C in a 5% CO2/95% air humidified incubator. Danu was dissolved in DMSO with a stock concentration of 50 mM, and was freshly diluted to the desired concentrations with culture medium. The final concentration of DMSO was at 0.05% (*v*/*v*).

### 4.3. Cell Viability Assay

The MTT assay was performed to examine the effect of Danu on the viability of C13 and A2780cp cells. Briefly, cells were seeded in 96-well culture plates at a density of 8 × l0^3^ cells/well. After cells were attached, the cells were treated with Danu at different concentrations (0.01–50 μM). The control cells received the vehicle only. After 24-h incubation, 10 μL MTT (5 g/L) was added to each well and cultured for another 4 h. Then, the media was carefully aspirated and 100 μL DMSO was added. The absorbance at the 450 nm wavelength was measured with a Synergy H4 Hybrid microplate reader (BioTek Inc., Winooski, VT, USA). The IC_50_ values were determined using the relative viability over Danu concentration curve using GraphPad Prism 6.0 (GraphPad Software, Inc., La Jolla, CA, USA).

### 4.4. Cell Cycle Analysis

The effect of Danu on cell cycle distribution was determined by flow cytometry. Briefly, C13 and A2780cp cells were treated with Danu at different concentrations of 0.01, 0.1, and 0.5 μM for 24 h. In separate experiments, C13 and A2780cp cells were treated with 0.5 μM Danu for 4, 8, 12, 24, 48, or 72 h. Cells were suspended, washed by PBS, centrifuged, and fixed in 70% ethanol at −20 °C overnight. Then, the cells were resuspended in 1 mL of PBS containing 1 mg/mL RNase A and 50 μg/mL PI. Cells were incubated in the dark for 30 min at room temperature. A total number of 2 × 10^4^ cells was subject to cell cycle analysis using a flow cytometer (Becton Dickinson Immunocytometry Systems, San Jose, CA, USA).

### 4.5. Quantification of Cellular Apoptosis

The effect of Danu on apoptosis of C13 and A2780cp cells was quantitated using the Annexin V: PE apoptosis detection kit (San Jose, CA, USA) according to the manufacturer’s instruction. Cells were treated with Danu at concentrations of 0.01, 0.1, and 0.5 μM for 48 h. The cells were resuspended at a concentration of 1 × 10^6^ cells/mL in 1× binding buffer. A quota of cell suspension (100 μL) was transferred into a clean 5 mL tube and incubated with 5 μL of Annexin V: PE and 5 μL of 7-amino-actinomycin D (a vital nucleic acid dye) in the dark for 15 min at room temperature. A quota of 1× binding buffer (400 μL) was then added to each tube, and the number of apoptotic cells was quantified using a flow cytometer within one hour. Cells that stain positive for Annexin V: PE and negative for 7-amino-actinomycin D are undergoing apoptosis, cells that stain positive for both Annexin V: PE and 7-amino-actinomycin D are either in the final stage of apoptosis, are undergoing necrosis, or are already dead, and cells that stain negative for both Annexin V: PE and 7-amino-actinomycin D are alive and not undergoing measurable apoptosis.

### 4.6. Quantification of Cellular Autophagy

To examine the effect of Danu on autophagy in C13 and A2780cp cells, cellular autophagy was detected using flow cytometry. Briefly, C13 and A2780cp cells were seeded in 60 mm Petri dishes for 24 h and the cells reached ~75% confluence, then treated with fresh medium alone, control vehicle alone (0.05% DMSO, *v*/*v*), or Danu (0.01, 0.1, and 0.5 µM) for 24 h. In separate experiments, C13 and A2780cp cells were treated with 0.5 μM Danu for 4, 8, 12, 24, 48, or 72 h. Following the Danu treatment, cells were resuspended in 250 μL of phenol red-free culture medium containing 5% FBS and 250 μL of the diluted Cyto-ID^®^ Green stain solution was added to each sample and mixed well. Cells were incubated for 30 min at 37 °C in the dark and then collected by centrifugation at 250× g for 3 min. The cell pellet was washed with 1× assay buffer in the Cyto-ID^®^ autophagy detection kit and resuspended in 500 μL fresh 1× assay buffer. Cells were analyzed using the green (FL1) channel of the flow cytometer.

### 4.7. Confocal Fluorescence Microscopy

The effect of Danu on cellular autophagy was further examined using confocal microscopy with the application of Cyto-ID^®^ autophagy detection kit according to the manufacture’s instruction. The C13 and A2780cp cells were seeded into 8-well chamber slide at 30% confluence. After incubation overnight, the cells were treated with Danu at 0.01, 0.1, and 0.5 μM. After incubation for 24 h, the cells reached ~60% of confluence and were washed with 1×assay buffer, following by incubation with 100 μL of microscopy dual detection reagent for 30 min at 37 °C in the dark. After incubation, the cells were washed with 1× assay buffer to remove the detection reagent, and then examined using a TCS SP2 laser scanning confocal microscope (Leica, Wetzlar, Germany) using a standard fluorescein isothiocyanate filter set for imaging the autophagic signal at wavelengths of 405/488 nm.

### 4.8. Western Blotting Analysis

C13 and A2780cp cells were washed with pre-cold PBS after 24-h treatment with Danu at 0.01, 0.1, and 0.5 μM, lysed with the RIPA buffer (50 mmol HEPES at pH 7.5, 150 mmol NaCl, 10% glycerol, 1.5 mmol MgCl_2_, 1% Triton-X 100, 1 mmol EDTA at pH 8.0, 10 mmol sodium pyrophosphate, 10 mmol sodium fluoride) containing the protease and phosphatase inhibitor cocktails, and centrifuged at 3000× *g* for 10 min at 4 °C. Protein concentrations were measured using Pierce BCA protein assay kit. Equal amount of protein sample (30 μg) was resolved by sodium dodecyl sulfate polyacrylamide gel electrophoresis (SDS-PAGE) sample loading buffer and electrophoresed on 7%–12% SDS-PAGE mini-gel after thermal denaturation at 95 °C for 5 min. Proteins were transferred onto PVDF membrane at 400 mA for 1–2 h at 4 °C. Membranes were probed with indicated primary antibody overnight at 4 °C and then blotted with respective secondary anti-mouse or anti-rabbit antibody. Visualization was performed using Bio-Rad ChemiDocTM XRS system (Hercules, CA, USA) with enhanced-chemiluminescence substrate and the blots were analyzed using Image Lab 3.0 (BioRad, Hercules, CA, USA). Protein level was normalized to the matching densitometry values of the internal control β-actin.

### 4.9. Statistical Analysis

Data are presented as the mean ± standard deviation (SD). Comparisons of multiple groups were evaluated by one-way analysis of variance (ANOVA) followed by Tukey’s multiple comparison procedure. Values of *p* < 0.05 were considered statistically significant. Assays were performed at least three times independently.

## Supplementary Materials

The following supporting information can be downloaded at: https://www.mdpi.com/article/10.3390/ijms161126018/s1.
