# Correction: Zi et al. Danusertib Induces Apoptosis, Cell Cycle Arrest, and Autophagy but Inhibits Epithelial to Mesenchymal Transition Involving PI3K/Akt/mTOR Signaling Pathway in Human Ovarian Cancer Cells. *Int. J. Mol. Sci.* 2015, *16*, 27228–27251

**DOI:** 10.3390/ijms262311467

**Published:** 2025-11-27

**Authors:** Dan Zi, Zhi-Wei Zhou, Ying-Jie Yang, Lin Huang, Zun-Lun Zhou, Shu-Ming He, Zhi-Xu He, Shu-Feng Zhou

**Affiliations:** 1 Department of Obstetrics and Gynecology, Affiliated Hospital of Guizhou Medical University, Guiyang 550004, China; 2 Department of Pharmaceutical Sciences, College of Pharmacy, University of South Florida, Tampa, FL 33612, USA; 3 Department of Gynecologic Oncology Surgery, Affiliated Cancer Hospital of Guizhou Medical University, Guiyang 550002, China; 4 Department of Obstetrics and Gynecology, Xiaolan Hospital, Southern Medical University, Zhongshan 528415, China; 5 Guizhou Provincial Key Laboratory for Regenerative Medicine, Stem Cell and Tissue Engineering Research Center & Sino-US Joint Laboratory for Medical Sciences, Guizhou Medical University, Guiyang 550004, China

In the original publication [1], there were errors in the p53 gel strip of A2780cp cells in Figure 4A and the C-caspase9 of A2780cp cells in Figure 7A, where both images were repeated. The p-Akt gel strip of C13 cells in Figure 11A and the p-mTOR of A2780 cells were repeated, as well as the p-mTOR gel strip of C13 cells and the Beclin-1. The Beclin-1 gel strip of A2780cp cells in Figure 11A was repeated with the E-cadherin of A2780cp cells in Figure 13A, as published. During the laboratory period, multiple people from the author’s experimental groups collaborated on the WB experiments, which led to errors in the labeling of the bands. The authors have found each original strip and made the corresponding corrections. The corrected Figure 7A, Figure 11A and Figure 13A appear below. The authors state that the scientific conclusions are unaffected. This correction was approved by the Academic Editor. The original publication has also been updated.

## Figures and Tables

**Figure 7:**
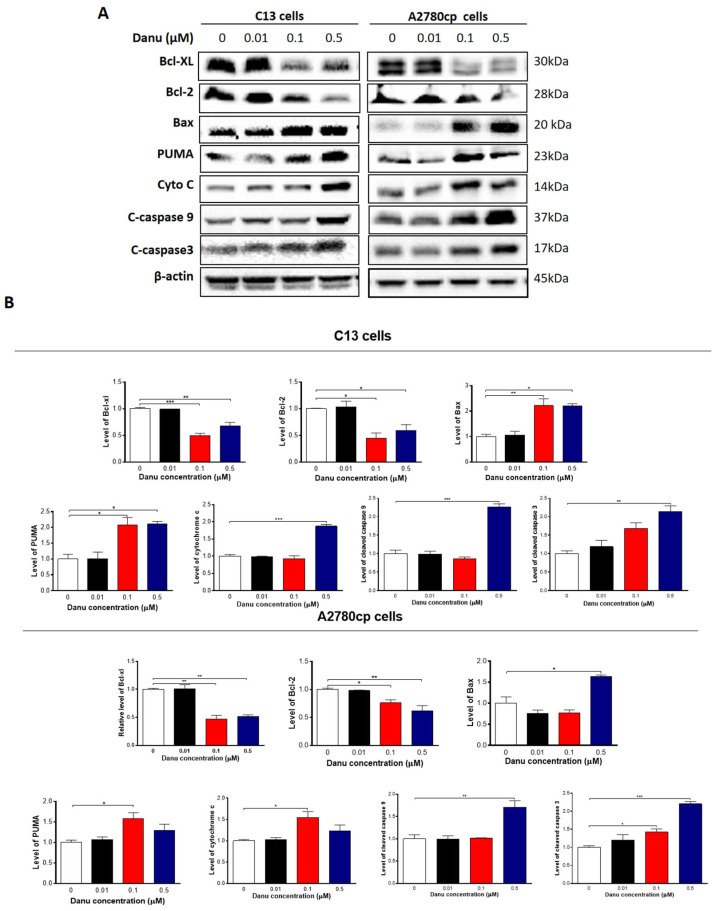
Danu alters the expression of pro-apoptotic and anti-apoptotic proteins in C13 and A2780cp cells. C13 and A2780cp cells were incubated with Danu 0.01, 0.1, and 0.5 μM for 48 h and the protein samples were subject to Western blotting assay. (**A**) Representative blots of B-cell lymphoma-extra-large (Bcl-xl), B-cell lymphoma 2 (Bcl-2), Bcl-2-like protein 4/Bcl-2-associated X protein (Bax), p53 up-regulated modulator of apoptosis (PUMA), cytochrome c, c-caspase 9, and c-caspase 3 in C13 and A2780cp cells. (**B**) Bar graphs showing relative expression level of Bcl-xl, Bcl-2, Bax, PUMA, cytochrome c, c-caspase 9, and c-caspase 3 in C13 and A2780cp cells. β-actin was used as the internal control. Data represent the mean ± SD of the three independent experiments. * *p* < 0.05, ** *p* < 0.01, and *** *p* < 0.001 by one-way analysis of variance.

**Figure 11:**
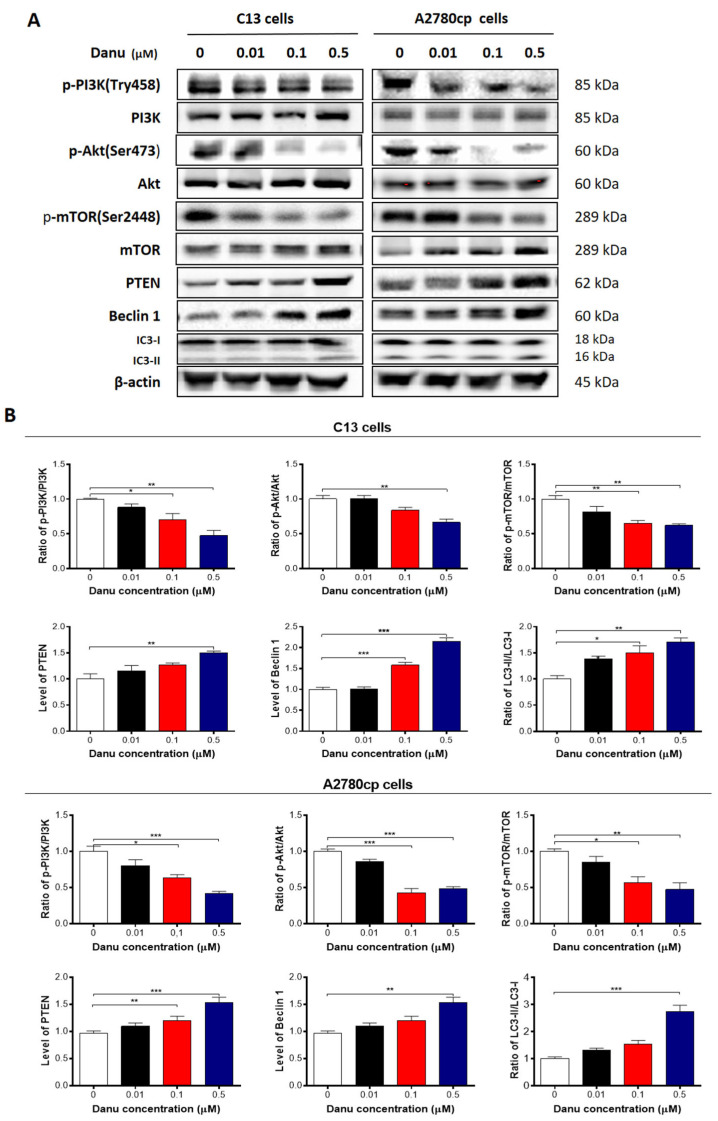
Danu alters the expression of pro-autophagic and anti-autophagic proteins in C13 and A2780cp cells. C13 and A2780cp cells were incubated with Danu 0.01, 0.1, and 0.5 μM for 24 h and the protein samples were subject to Western blotting assay. (**A**) Representative blots of phosphorylation level of PI3K, Akt, and mTOR, and the total level of PI3K, Akt, mTOR, PTEN, beclin 1, LC 3-I, and LC 3-II in C13 and A2780cp cells. (**B**) Bar graphs showing the ratio of p-PI3K/PI3K, p-Akt/Akt, and p-mTOR/mTOR, and expression of PTEN, beclin 1, LC 3-I, and LC 3-II in C13 and A2780cp cells. β-actin was used as the internal control. Data represent the mean ± SD of the three independent experiments. * *p* < 0.05, ** *p* < 0.01, and *** *p* < 0.001 by one-way analysis of variance.

**Figure 13:**
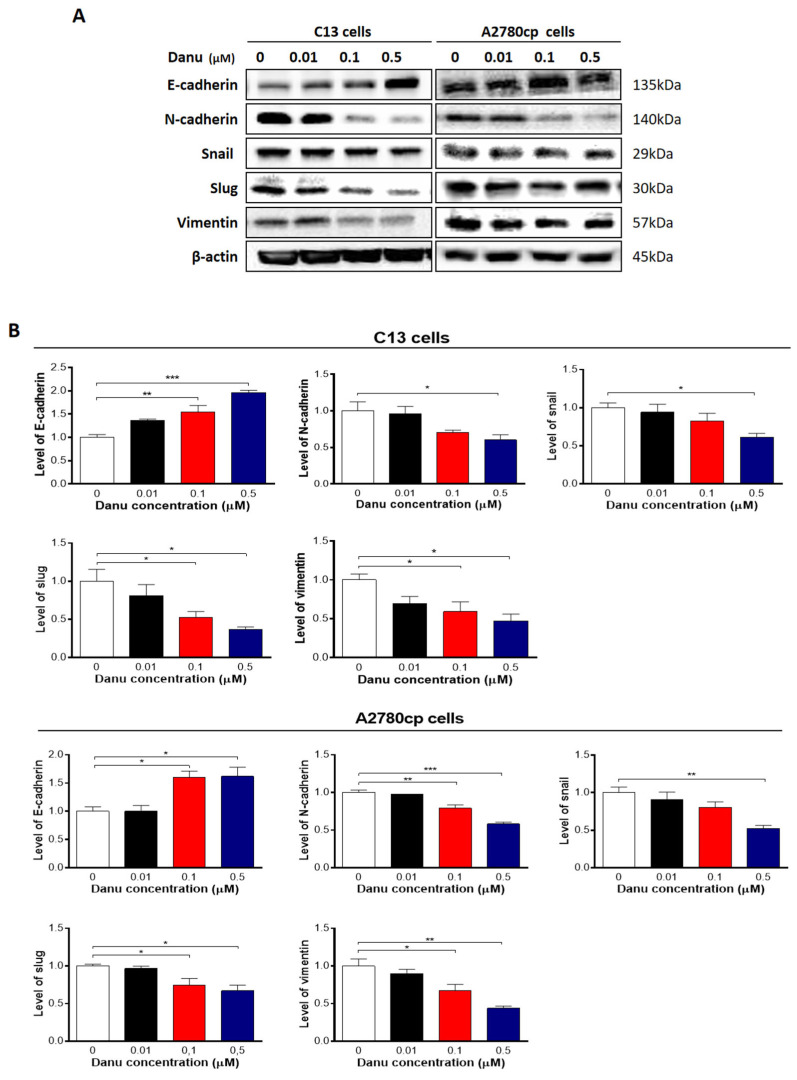
Danu inhibits EMT in C13 and A2780cp cells. C13 and A2780cp cells were incubated with Danu 0.01, 0.1, and 0.5 μM for 24 h and the protein samples were subject to Western blotting assay. (**A**) Representative blots showing E-cadherin, N-cadherin, snail, slug, and vimentin levels in C13 and A2780cp cells. (**B**) Bar graphs showing the relative expression of E-cadherin, N-cadherin, snail, slug, and vimentin in C13 and A2780cp cells. β-actin was used as the internal control. Data represent the mean ± SD of the three independent experiments. * *p* < 0.05, ** *p* < 0.01, and *** *p* < 0.001 by one-way analysis of variance.
