# Correction: Zi et al. Danusertib Induces Apoptosis, Cell Cycle Arrest, and Autophagy but Inhibits Epithelial to Mesenchymal Transition Involving PI3K/Akt/mTOR Signaling Pathway in Human Ovarian Cancer Cells. *Int. J. Mol. Sci.* 2015, *16*, 27228–27251

**DOI:** 10.3390/ijms27083495

**Published:** 2026-04-14

**Authors:** Dan Zi, Zhi-Wei Zhou, Ying-Jie Yang, Lin Huang, Zun-Lun Zhou, Shu-Ming He, Zhi-Xu He, Shu-Feng Zhou

**Affiliations:** 1 Department of Obstetrics and Gynecology, Affiliated Hospital of Guizhou Medical University, Guiyang 550004, China; zidangy08@163.com (D.Z.); yyjgmc@163.com (Y.-J.Y.); hllgmc@163.com (L.H.); zzlgmc@163.com (Z.-L.Z.); 2 Department of Pharmaceutical Sciences, College of Pharmacy, University of South Florida, Tampa, FL 33612, USA; zhouzw105@outlook.com; 3 Department of Gynecologic Oncology Surgery, Affiliated Cancer Hospital of Guizhou Medical University, Guiyang 550002, China; 4 Department of Obstetrics and Gynecology, Xiaolan Hospital, Southern Medical University, Zhongshan 528415, China; hsmgmc@163.com; 5 Guizhou Provincial Key Laboratory for Regenerative Medicine, Stem Cell and Tissue Engineering Research Center & Sino-US Joint Laboratory for Medical Sciences, Guizhou Medical University, Guiyang 550004, China

**1. Supplementary Material**

The authors would like to publish the raw data for Figures 7, 11, and 13 in the original publication [1] as Supplementary Materials. The citation of Supplementary Materials has been added in the caption of Figures 7, 11 and 13. **Supplementary Materials:** The following supporting information can be downloaded at: https://www.mdpi.com/article/10.3390/ijms161126018/s1.

**2. Text Correction**

There were typographical errors in Sections 2.2 and 2.4. Due to a typing mistake, an extra decimal point was added: 93.4.5% should actually be 93.5%, and 11.4.2% should be 11.4%.

A correction has been made to Sections 2.2 and 2.4:

2.2. Danu Induces Cell Cycle Arrest in G2/M Phase in C13 and A2780cp Cells

Notably, there was an evident occurrence of polyploidy in C13 and A2780cp cells when cells were treated with 0.5 µM Danu from 4 to 72 h. With 4-, 8-, 12-, 24-, 48- to 72-h treatment, the percentage of polyploidy of C13 cells was increased from 8.9%, 14.4%, 31.3%, 63.8%, 68.9% to 72.2%, correspondently, the percentage of diploidy was decreased from 91.1%, 85.6%, 68.7%, 36.2%, 31.1% to 27.8% (Figure 3B). Similarly, the percentage of polyploidy of A2780cp cells was increased from 2.8%, 6.6%, 13.1%, 33.7%, 70.0% to 87.4%, correspondently, the percentage of diploidy was decreased from 7.2%, 93.5%, 86.9%, 66.3%, 30.0% to 12.6% (Figure 3B). Collectively, these results demonstrate that Danu exerts a potent cell cycle arresting effect in C13 and A2780cp cells.

2.4. Danu Induces Apoptosis of C13 and A2780cp Cells via Activation of Mitochondria-Dependent Pathway

To further examine the cancer cell killing effect of Danu on C13 and A2780cp cells, the effect of Danu on apoptosis was tested by flow cytometry. Exposure of cells to Danu for 48 h resulted in a remarkable apoptosis of C13 and A2780cp cells (Figures 5 and 6). In C13 cells, the total percentage of apoptotic cells (early + late apoptosis) was 3.2%, 8.0%, and 37.4% when treated with Danu at 0.01, 0.1, and 0.5 μM for 48 h, respectively (Figure 5A,B). There was a 2.6- and 11.9-fold increase when treated with 0.1 and 0.5 μM Danu, compared to the control cells (*p* < 0.001; Figure 5A,B). Treating A2780cp cells with Danu 0.1 and 0.5 μM for 48 h increased the total percentage of apoptotic cells (early and late apoptosis) from 3.2% at the basal level to 32.4% and 49.2%, and there was a 10.1-, and 15.4-fold increase in apoptotic A2780cp cells, compared with the control, respectively (*p* < 0.001, Figure 5A,B). In addition, the effect of Danu on the apoptosis of C13 and A2780cp cells was examined when the cells were treated over 72 h. Incubation of C13 and A2780cp cells with 0.5 μM Danu time-dependently increased the number of apoptotic cells (Figure 6A,B). The percentage of apoptotic C13 cells was increased from 8.2% at basal level (zero time) to 11.6%, 11.4%, 11.0%, 14.5%, 19.9%, and 32.0% when treated with 0.5 μM Danu for 4, 8, 12, 24, 48, and 72 h, respectively; and there was a 2.4- and 3.9-fold rise in the apoptotic C13 cells after 48- and 72-h treatment, respectively (*p* < 0.01 or 0.001; Figure 6A,B). Similarly, the percentage of apoptotic A2780cp cells was increased from 2.2% at basal level to 3.1%, 3.3%, 5.4%, 17.3%, and 34.1% when treated with 0.5 µM Danu for 4, 12, 24, 48 and 72 h, respectively; and there was a 7.9- and 15.5-fold elevation in the apoptotic A2780cp cells after 48- and 72-h treatment, respectively (*p* < 0.001; Figure 6A,B). In aggregate, Danu remarkably induces apoptotic cell death in both C13 and A2780cp cells.

The authors state that the scientific conclusions are unaffected. This correction was approved by the Academic Editor. The original publication has also been updated.
