# Supplementary material for: Danusertib Induces Apoptosis, Cell Cycle Arrest, and Autophagy but Inhibits Epithelial to Mesenchymal Transition Involving PI3K/Akt/mTOR Signaling Pathway in Human Ovarian Cancer Cells
Source: Int J Mol Sci. 2015 Nov 13;16(11):27228–51. doi: 10.3390/ijms161126018 (PMC4661876; doi:10.3390/ijms161126018)
Supplement: Supplementary file 1 [file ijms-16-26018-s001.zip › Revised data and original strips 2025.10.11/Figure 11(Revised).pptx]

## Slide 1
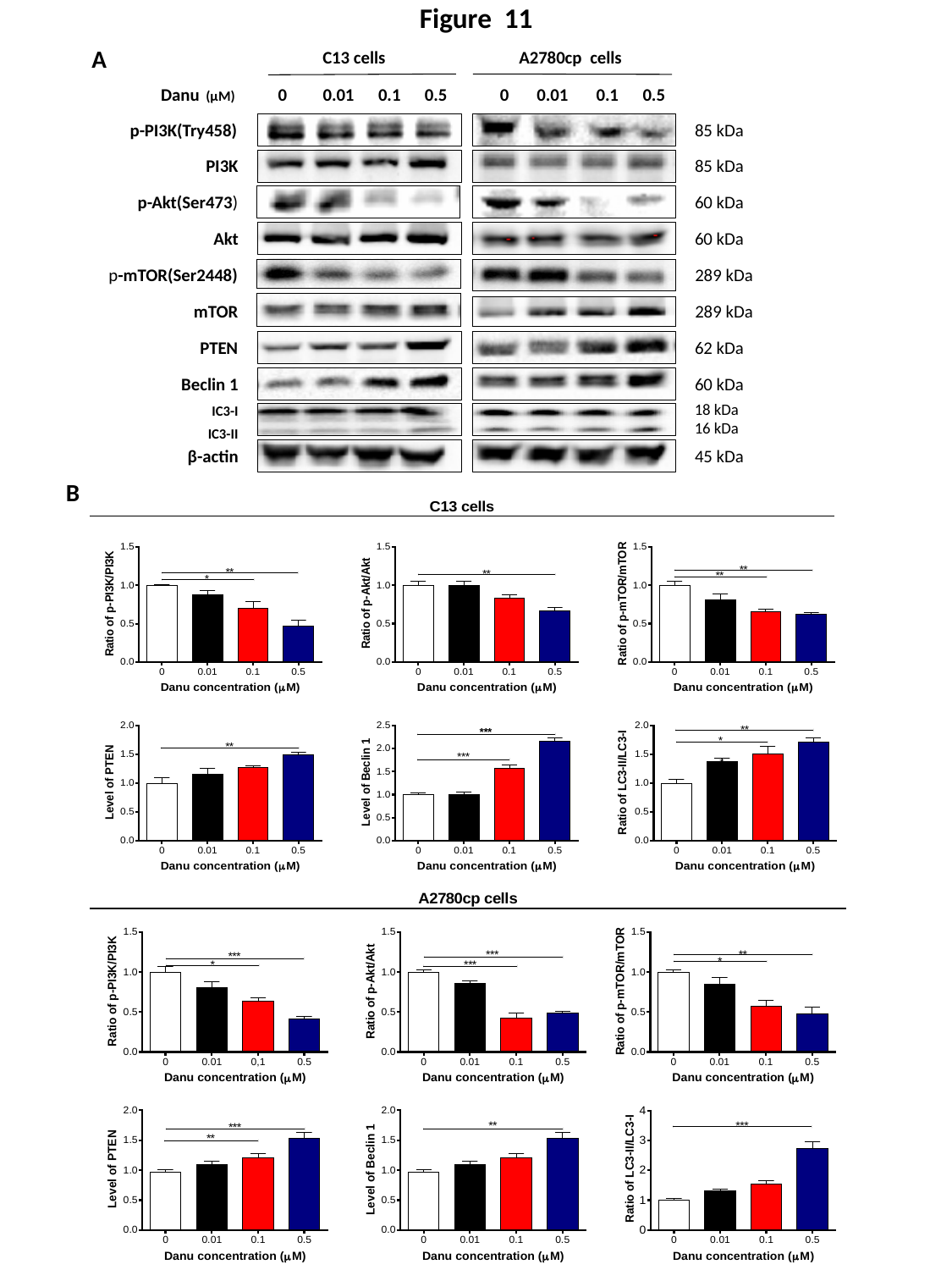

Figure 11
A
A2780cp cells
C13 cells
Danu (μM)
0 0.01 0.1 0.5
0 0.01 0.1 0.5
p-PI3K(Try458)
85 kDa
PI3K
85 kDa
p-Akt(Ser473)
60 kDa
Akt
60 kDa
 p-mTOR(Ser2448)
289 kDa
mTOR
289 kDa
PTEN
62 kDa
Beclin 1
60 kDa
IC3-I
IC3-II
18 kDa
16 kDa
β-actin
45 kDa
B
