# Supplementary material for: Danusertib Induces Apoptosis, Cell Cycle Arrest, and Autophagy but Inhibits Epithelial to Mesenchymal Transition Involving PI3K/Akt/mTOR Signaling Pathway in Human Ovarian Cancer Cells
Source: Int J Mol Sci. 2015 Nov 13;16(11):27228–51. doi: 10.3390/ijms161126018 (PMC4661876; doi:10.3390/ijms161126018)
Supplement: Supplementary file 1 [file ijms-16-26018-s001.zip › Revised data and original strips 2025.10.11/Figure 13(new).pptx]

## Slide 1
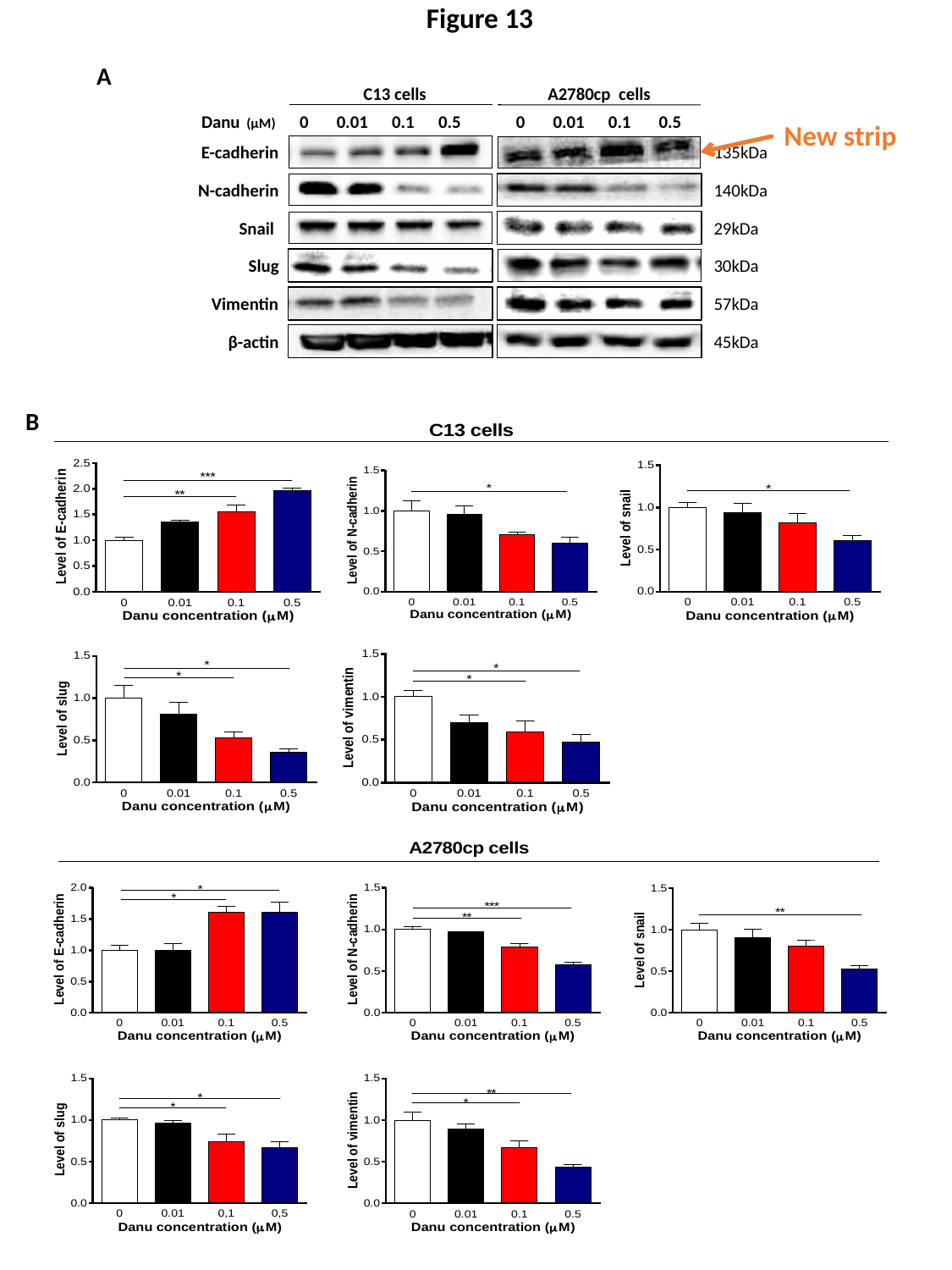

Figure 13
A
 C13 cells
A2780cp cells
Danu (μM)
0 0.01 0.1 0.5
0 0.01 0.1 0.5
New strip
E-cadherin
135kDa
N-cadherin
140kDa
Snail
29kDa
 Slug
30kDa
Vimentin
57kDa
β-actin
45kDa
B
