# Supplementary material for: Danusertib Induces Apoptosis, Cell Cycle Arrest, and Autophagy but Inhibits Epithelial to Mesenchymal Transition Involving PI3K/Akt/mTOR Signaling Pathway in Human Ovarian Cancer Cells
Source: Int J Mol Sci. 2015 Nov 13;16(11):27228–51. doi: 10.3390/ijms161126018 (PMC4661876; doi:10.3390/ijms161126018)
Supplement: Supplementary file 1 [file ijms-16-26018-s001.zip › Revised data and original strips 2025.10.11/Figure 7(Revised).pptx]

## Slide 1
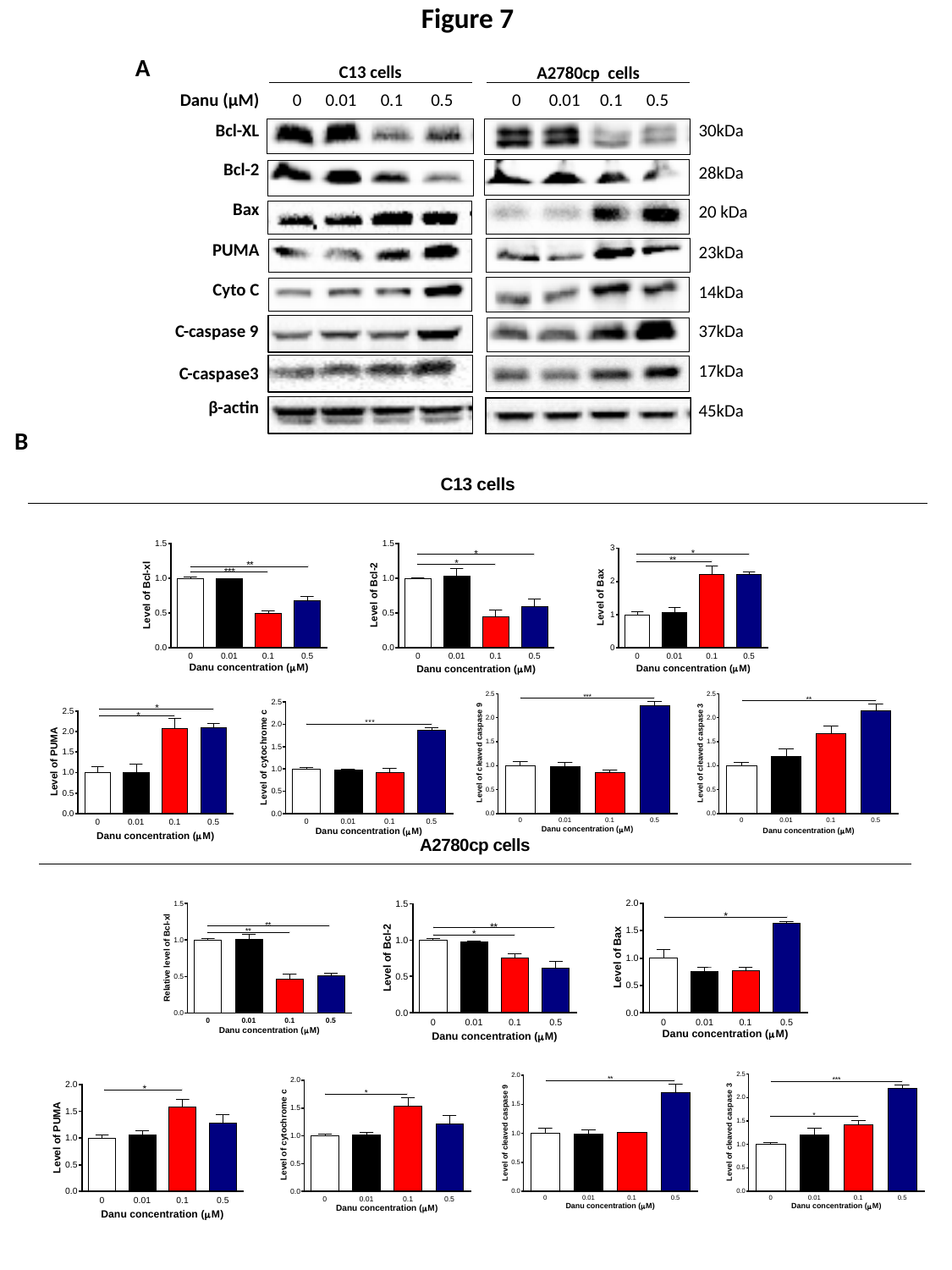

Figure 7
A
C13 cells
A2780cp cells
Danu (μM)
 0 0.01 0.1 0.5
 0 0.01 0.1 0.5
Bcl-XL
30kDa
Bcl-2
28kDa
Bax
20 kDa
PUMA
23kDa
Cyto C
14kDa
C-caspase 9
37kDa
17kDa
 C-caspase3
β-actin
45kDa
B
