# Supplementary figures and images for: Danusertib Induces Apoptosis, Cell Cycle Arrest, and Autophagy but Inhibits Epithelial to Mesenchymal Transition Involving PI3K/Akt/mTOR Signaling Pathway in Human Ovarian Cancer Cells
Source: Int J Mol Sci. 2015 Nov 13;16(11):27228–51. doi: 10.3390/ijms161126018 (PMC4661876; doi:10.3390/ijms161126018)

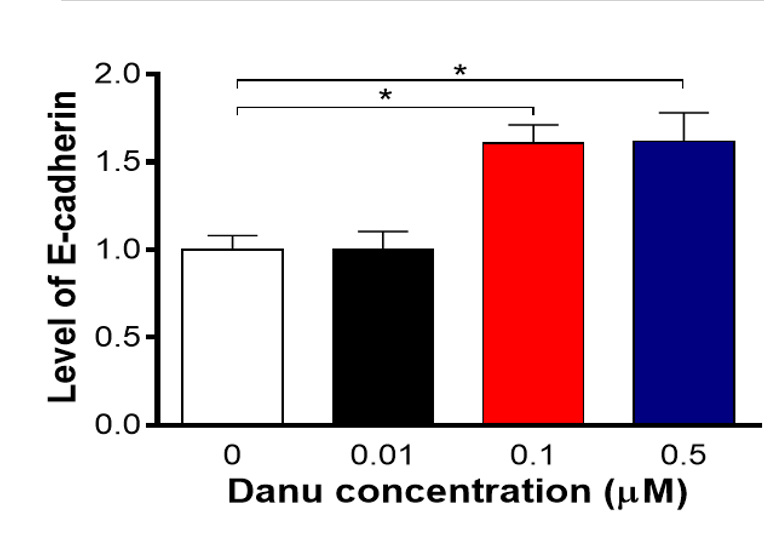

Supplement: Supplementary file 1 [file ijms-16-26018-s001.zip › Raw data of Figure 13/A2780cp Cells/E-cadherin.png]

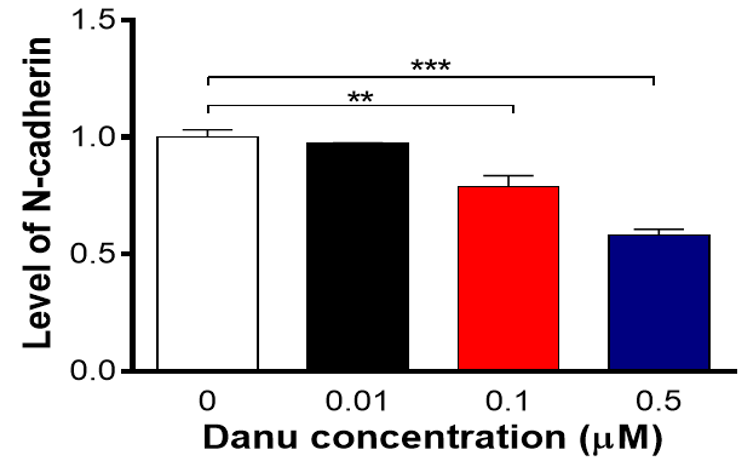

Supplement: Supplementary file 1 [file ijms-16-26018-s001.zip › Raw data of Figure 13/A2780cp Cells/N-cadherin.png]

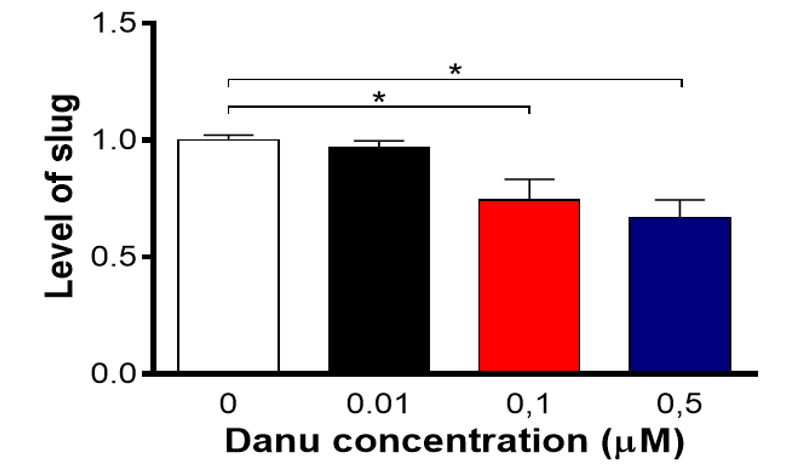

Supplement: Supplementary file 1 [file ijms-16-26018-s001.zip › Raw data of Figure 13/A2780cp Cells/Slug.png]

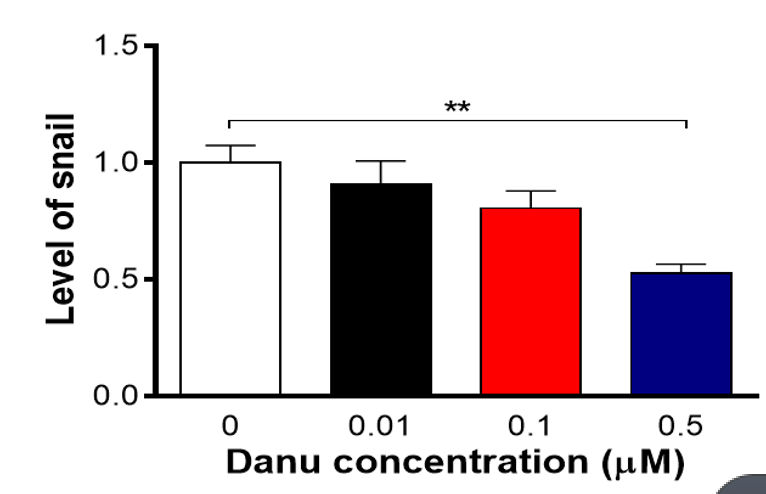

Supplement: Supplementary file 1 [file ijms-16-26018-s001.zip › Raw data of Figure 13/A2780cp Cells/Snail.png]

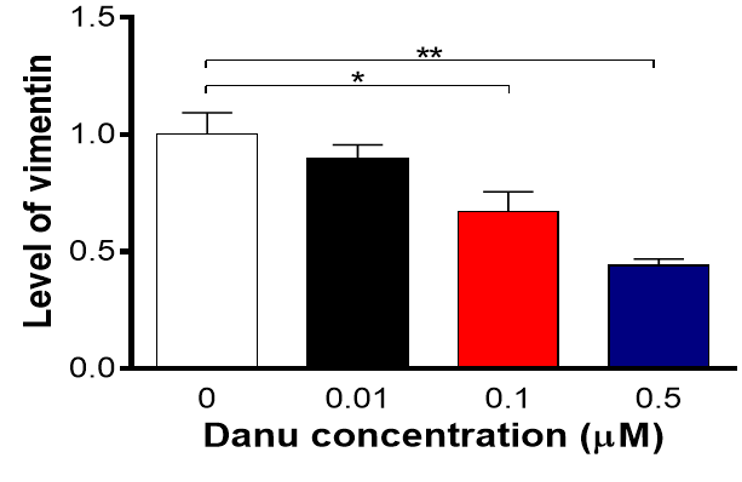

Supplement: Supplementary file 1 [file ijms-16-26018-s001.zip › Raw data of Figure 13/A2780cp Cells/Vimentin.png]

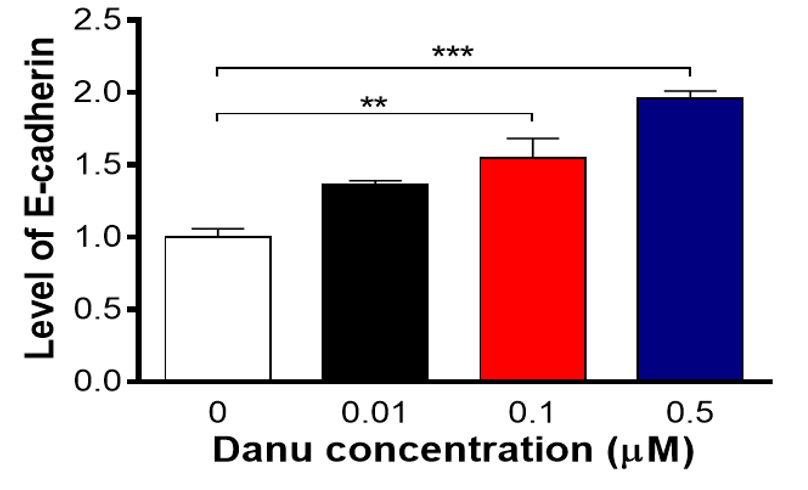

Supplement: Supplementary file 1 [file ijms-16-26018-s001.zip › Raw data of Figure 13/C13 cells/E-cadherin .png]

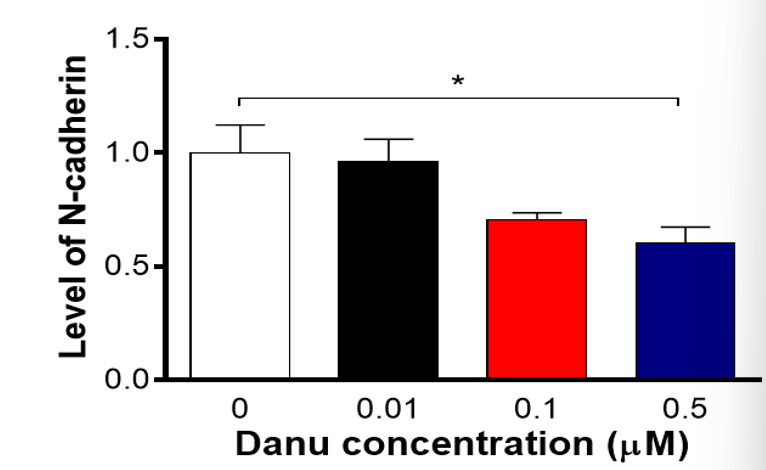

Supplement: Supplementary file 1 [file ijms-16-26018-s001.zip › Raw data of Figure 13/C13 cells/N-cadherin.png]

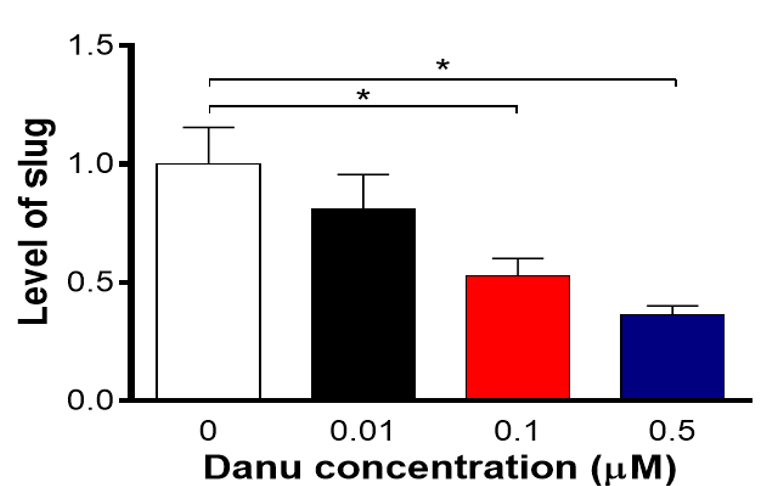

Supplement: Supplementary file 1 [file ijms-16-26018-s001.zip › Raw data of Figure 13/C13 cells/Slug.png]

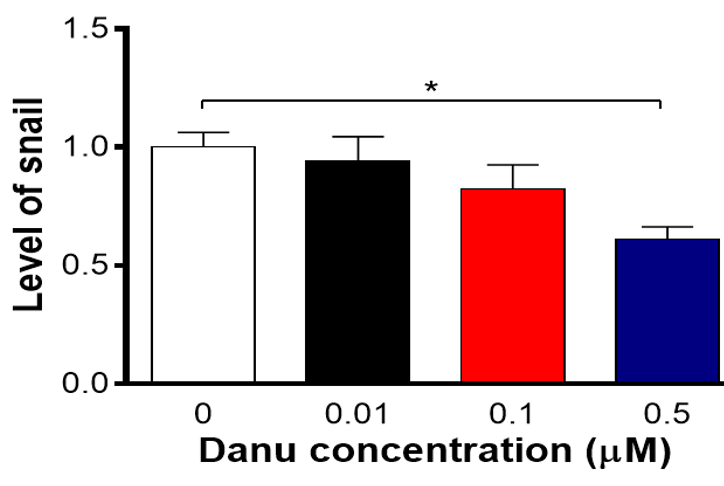

Supplement: Supplementary file 1 [file ijms-16-26018-s001.zip › Raw data of Figure 13/C13 cells/Snail.png]

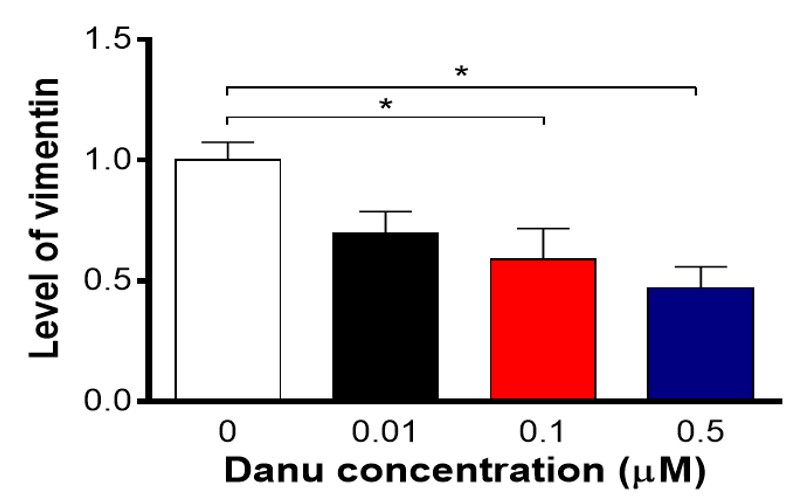

Supplement: Supplementary file 1 [file ijms-16-26018-s001.zip › Raw data of Figure 13/C13 cells/Vimentin.png]

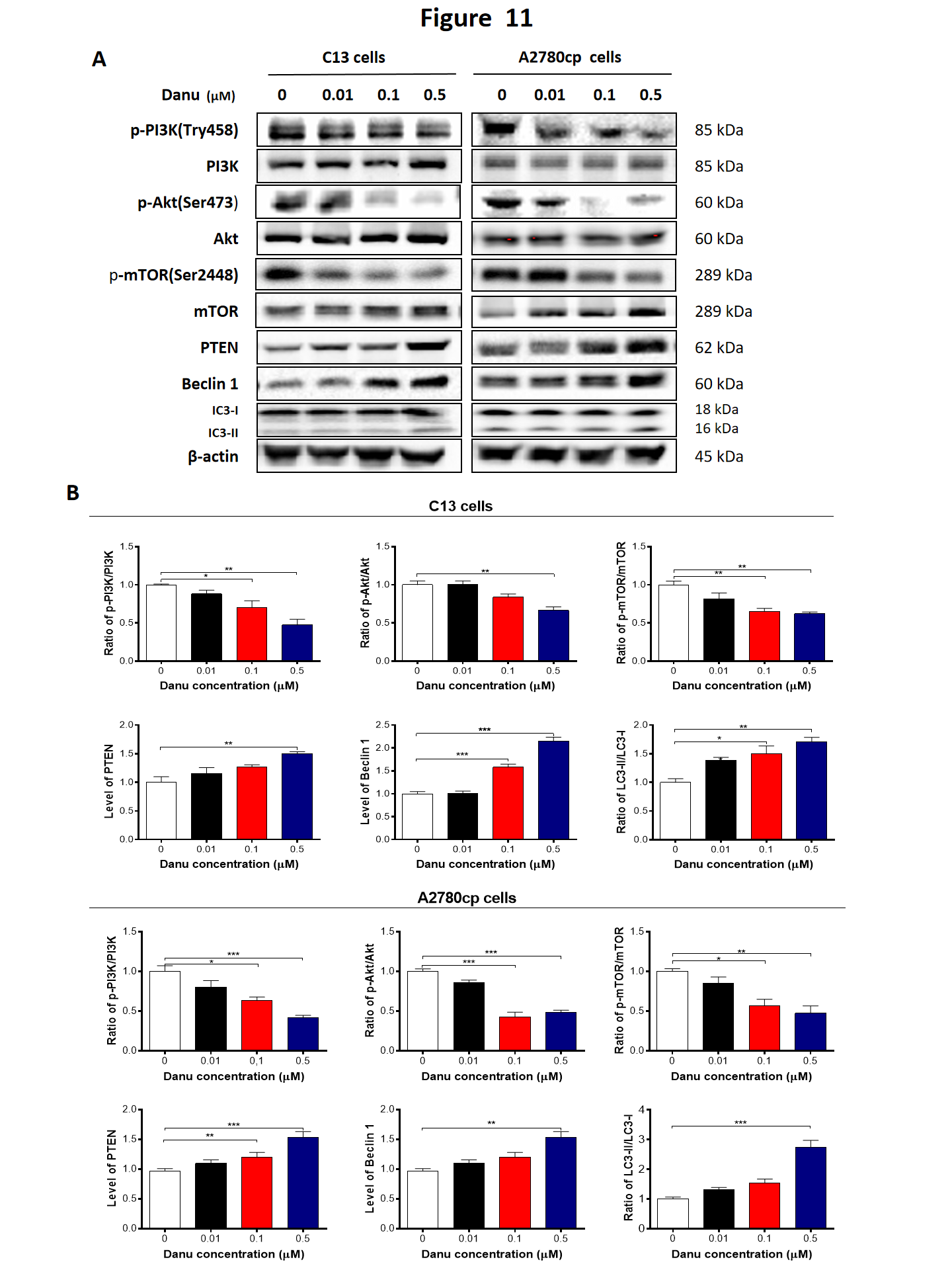

Supplement: Supplementary file 1 [file ijms-16-26018-s001.zip › Revised data and original strips 2025.10.11/Figure 11(Revised).tif]

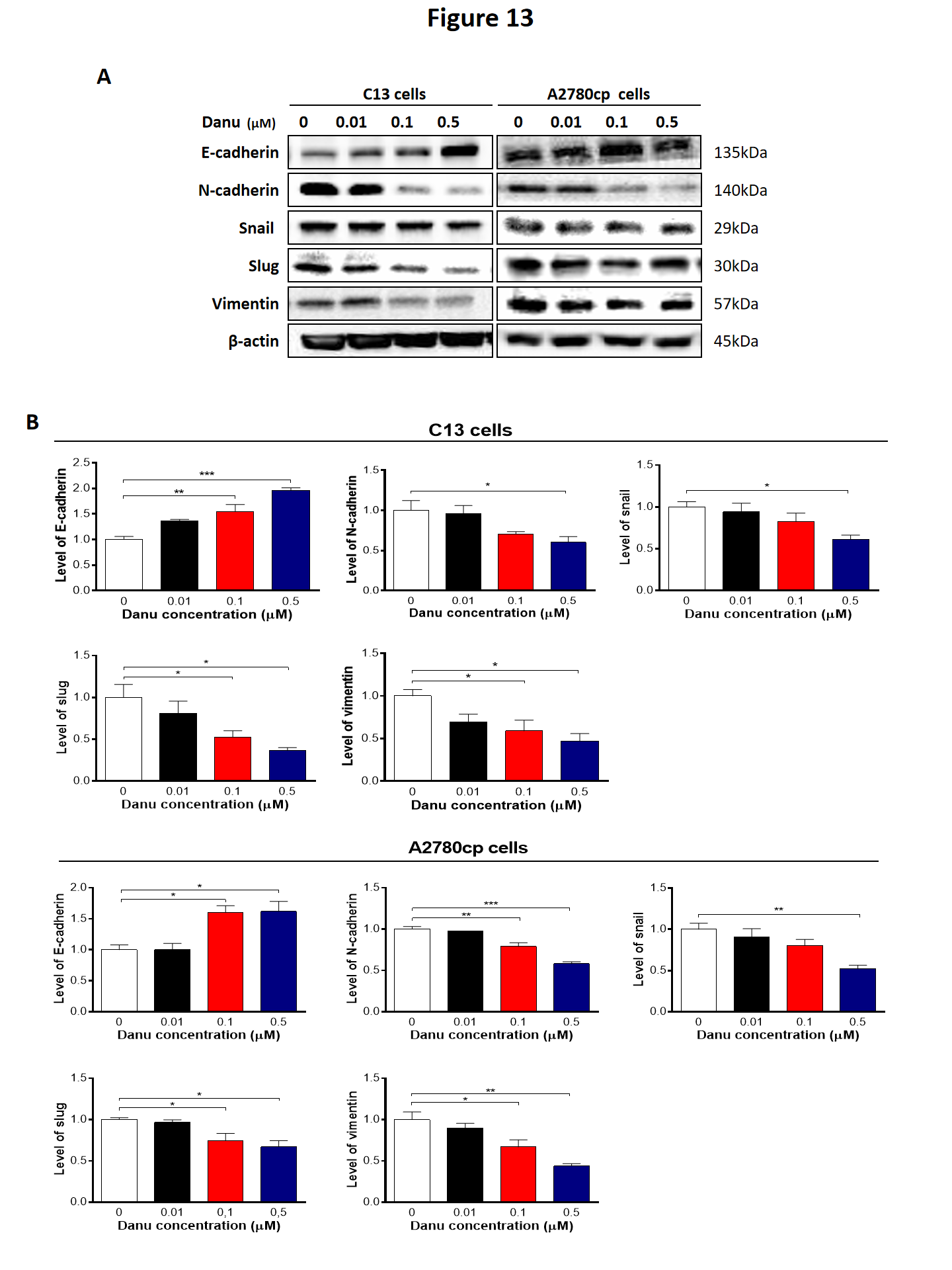

Supplement: Supplementary file 1 [file ijms-16-26018-s001.zip › Revised data and original strips 2025.10.11/Figure 13(Revised).tif]

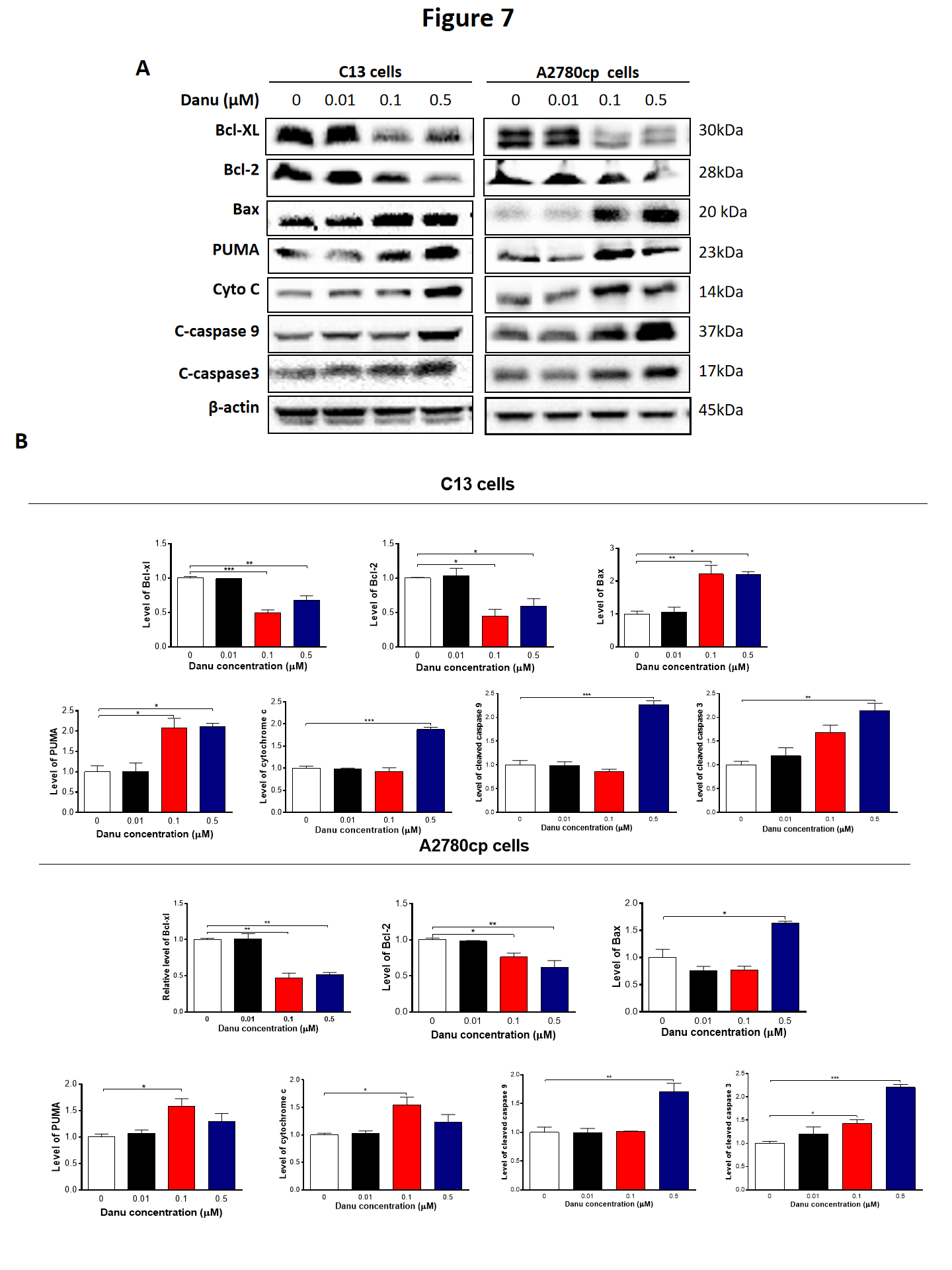

Supplement: Supplementary file 1 [file ijms-16-26018-s001.zip › Revised data and original strips 2025.10.11/Figure 7(Revised).tif]

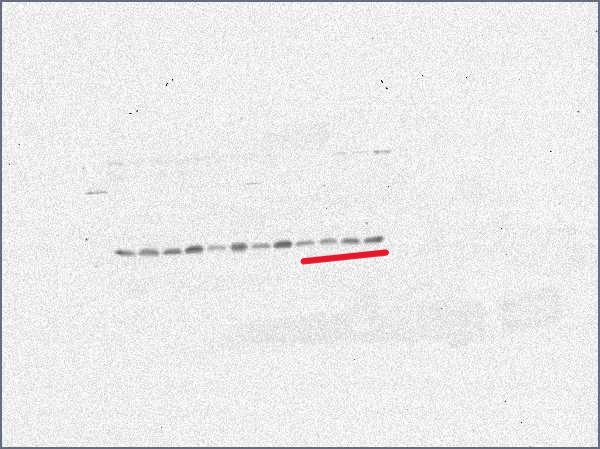

Supplement: Supplementary file 1 [file ijms-16-26018-s001.zip › Revised data and original strips 2025.10.11/Original strips/green strips/Figure11A C13- bclin-1.jpg]

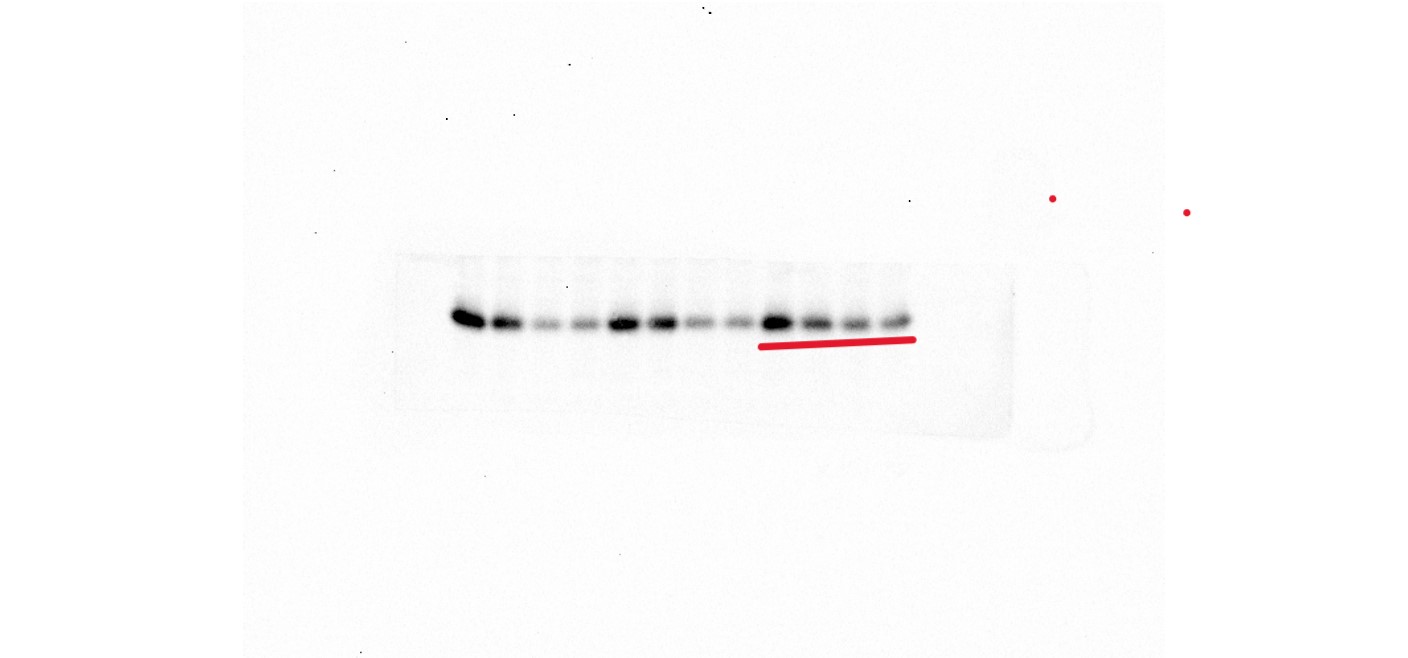

Supplement: Supplementary file 1 [file ijms-16-26018-s001.zip › Revised data and original strips 2025.10.11/Original strips/green strips/Figure11A-C13 p-mTOR(Replacement).jpg]

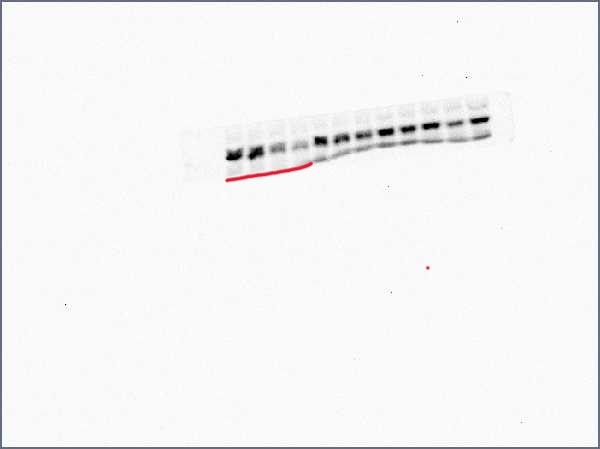

Supplement: Supplementary file 1 [file ijms-16-26018-s001.zip › Revised data and original strips 2025.10.11/Original strips/pink strips/Figure11A C13-P-AKT.jpg]

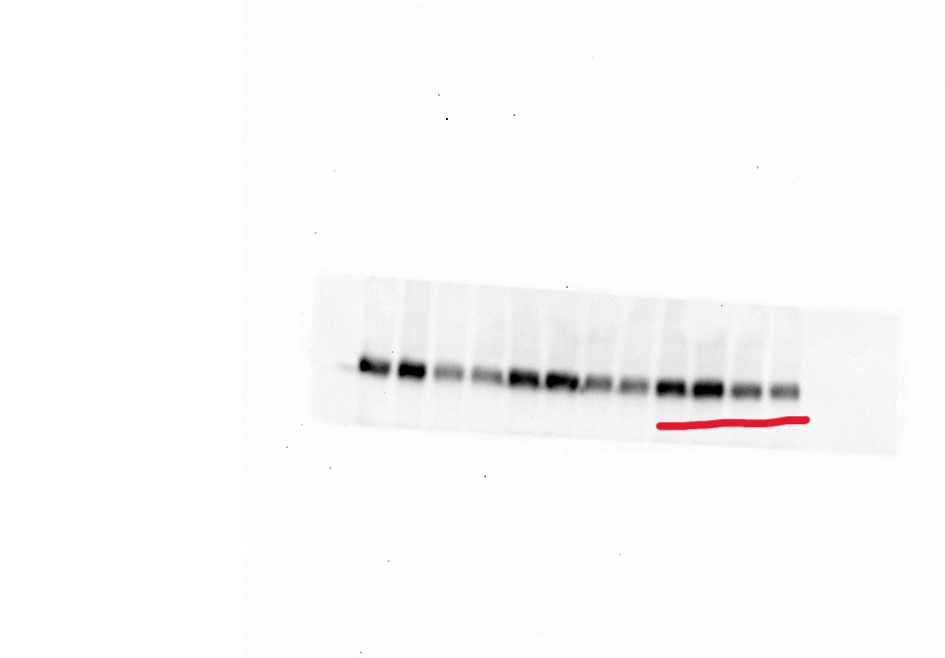

Supplement: Supplementary file 1 [file ijms-16-26018-s001.zip › Revised data and original strips 2025.10.11/Original strips/pink strips/Figure11A A2780cp p-mTOR(replacement).jpg]

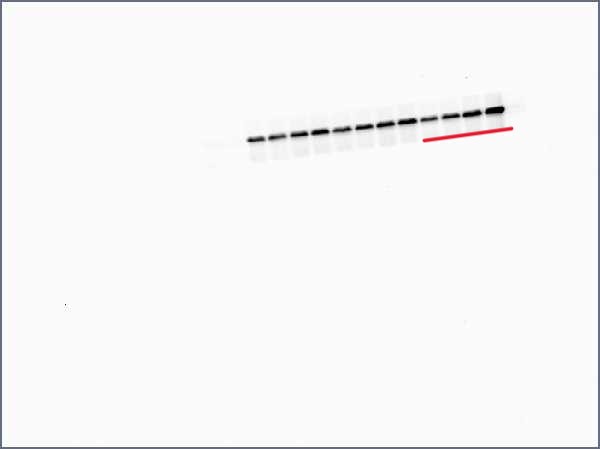

Supplement: Supplementary file 1 [file ijms-16-26018-s001.zip › Revised data and original strips 2025.10.11/Original strips/red strips/Figure4 A2780cp- P53.jpg]

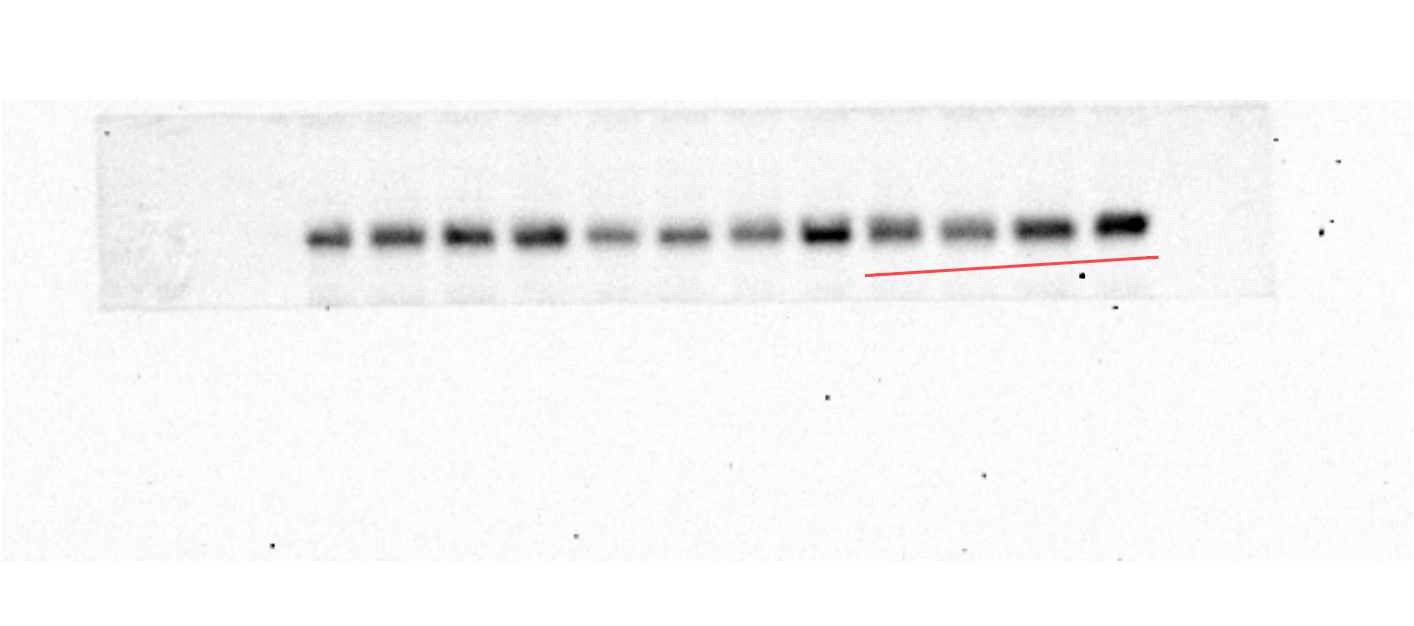

Supplement: Supplementary file 1 [file ijms-16-26018-s001.zip › Revised data and original strips 2025.10.11/Original strips/red strips/Figure7A A2780cp C-caspase3(Replacement)1 - 副本.png]

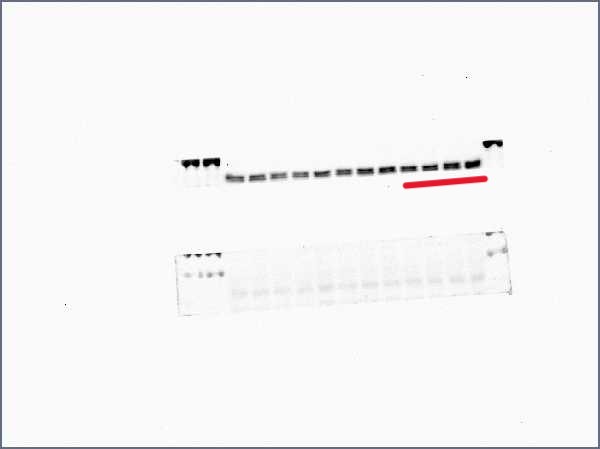

Supplement: Supplementary file 1 [file ijms-16-26018-s001.zip › Revised data and original strips 2025.10.11/Original strips/yellow strips/Figure 11A up-A2780-Beclin-1.jpg]

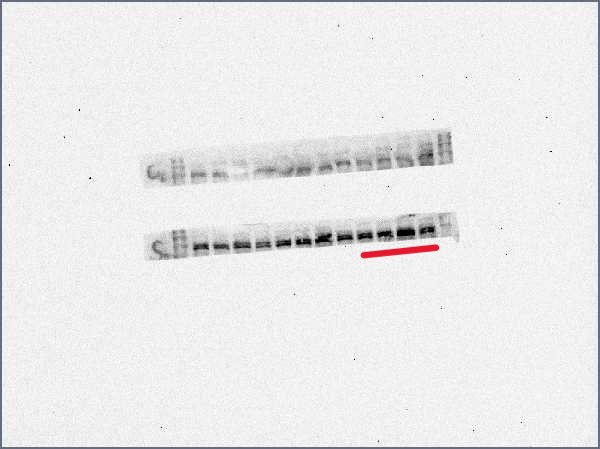

Supplement: Supplementary file 1 [file ijms-16-26018-s001.zip › Revised data and original strips 2025.10.11/Original strips/yellow strips/Figure13A down-A2780cp E-cadherin(Replacement).jpg]
